# Pollinating fig wasps’ simple solutions to complex sex ratio problems: a review

**DOI:** 10.1186/s12983-021-00447-4

**Published:** 2022-01-12

**Authors:** Jaco M. Greeff, Finn Kjellberg

**Affiliations:** 1 Department of Biochemistry, Genetics and Microbiology, University of Pretoria, Pretoria, South Africa; 2 CEFE, CNRS, Univ Montpellier, EPHE, IRD, Montpellier, France

**Keywords:** Local mate competition, Sex allocation, Adaptation, Pre-adaptation, Exaptation, Mortality, Agaonidae, *Ficus*, Hymenoptera

## Abstract

Local mate competition (LMC) favours female biased clutch sex ratios because it reduces competition between brothers and provides extra mating opportunities for sons. Fig wasps seem to fit LMC model assumptions and lay female-biased sex ratios as predicted. These female biased sex ratios increase fitness greatly. In line with predictions, their sex ratios become less female-biased as the number of mothers laying in the same fig increases. However, this variation results in comparatively small fitness benefits compared to just biased ratios and data suggest substantial mismatches with LMC theory. The mismatches are due to several factors. (1) Multiple foundresses typically lay too many daughters. (2) Single foundress sex ratios are explained by sequential oviposition and ladies-last models. (3) Mortality that typically exceeds 10% may decouple the link between primary sex ratios, the focus of model predictions, and secondary sex ratios of adult wasps that are counted by researchers. (4) Model assumptions are frequently violated: (a) clutch sizes are unequal, (b) oviposition may not be simultaneous (c) cryptic/multiple wasp species inhabit the same host, (d) foundress numbers are systematically undercounted, (e) inbreeding coefficient calculations are inaccurate, and (f) male wasps sometimes disperse. These data and calculations suggest that alternative explanations must be considered seriously. Substantial data show that wasps typically lay most of their male eggs first followed by mostly female eggs require a new approach. These “slope” strategies result in more accurate sex ratios that are automatically adjusted to foundress number, own and relative clutch sizes and to sequential clutches. This effect will alter sex ratios in all species once the egg capacity of a fig is crossed or when interference reduces clutch sizes. In addition to this passive response, the females of about half the studied species have a conditional response that reduces female bias under higher foundress numbers by laying more sons. Therefore, wasps seem to use a very simple strategy that increases their fitness. Natural selection could have optimized parameters of the slope strategy and possibly the existence of the slope strategy itself. Variation in the slope strategy that is the result of natural selection is adaptive. Research should therefore focus on quantifying variables of this slope strategy. Currently, it is unclear how much of the variation is adaptive as opposed to being coincidental by-products.

**Supplementary Information:**

The online version contains supplementary material available at 10.1186/s12983-021-00447-4.

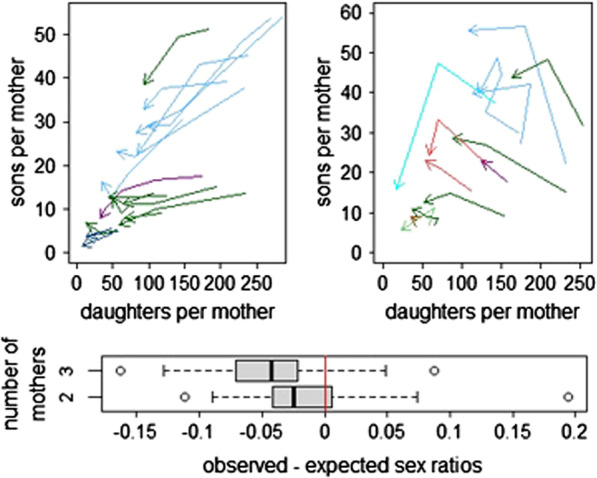

“Reading about them was not enough, they were too fantastic” Hamilton writing about fig wasps [1]

## Introduction

The close fit between sex ratios and optimality predictions is regarded as a triumph of the demonstration of the role of natural selection in evolution [1–5]. This is somewhat ironic as Darwin could not explain the adaptive benefits of sex ratios, although he came close [6]. It is claimed that the sex ratio adjustment of pollinating fig wasps (Agaonidae) offers some of the most convincing evidence for the role of natural selection [2, 7]. However, Orzack pointed out that taking mean sex ratios rather than determining individual females’ sex ratio strategies is a major shortcoming in many fig wasp sex ratio studies [8, 9]. Taking means results in models' predictions being tested at a different causal level from that at which individual selection operates [10]. A mean sex ratio obscures any heterogeneity of sex ratios within the group and can at most support the claim that the group produces an optimal sex ratio. However, optimality models of sex ratio behaviours predict the sex ratio(s) produced by individuals and are based on the assumption that every mother follows the optimal sex ratio strategy. Accordingly, testing the claim that sex ratios are optimal requires data on individuals. Furthermore, heritable variation in sex ratios would mean that an optimal genotype has not been fixed in the population [11, 12] and no fig wasp study has investigated the heritability of variation in sex ratios. We will argue that the claims of a close fit are exaggerated and that the subtlety of sex ratio adjustments is overstated.

In this review we introduce local mate competition (LMC). We show that it results in female-biased sex ratios and that the fitness consequence of biased sex ratios is substantial. In contrast, the fitness benefits of small conditional adjustments are much smaller. We consider the effect of sex ratio precision on fitness and how it manifests in pollinating fig wasps. We consider the role and magnitude of developmental mortality that potentially decouples sex ratios of eggs from that of adults. We explain why fig wasps were considered a good model system and the experimental approaches that are used to understand their sex ratio variation. Next, we question the good fit between theory and sex ratio data and model assumptions. We give alternative explanations for single and multi-foundress sex ratios. We argue for the importance of fieldwork that focuses on the variability in wasp biology and on the actual traits under selection, rather than on the sex ratio that is an emergent property.

## Local mate competition

Local Mate Competition (LMC) occurs when one or a few females lay their eggs in a patch and matings occur among brood members before offspring disperse [13]. This means that mating is not panmictic but restricted to family groups. Hamilton calculated the “unbeatable sex ratio”, the selected proportion of sons in a clutch, under LMC conditions [13]. It predicts female-biased sex ratios because exchanging sons for daughters reduces the waste of energy on sons that frequently compete with their brothers and increases the number of mating opportunities to the remaining sons [13, 14]. Many empirical studies support the theory that sex ratios are female-biased when LMC occurs [4] (note that some of these examples are unconvincing [9]).

Many of the species that bias their sex ratios are haplodiploid. Haplodiploidy allows a proximate mechanism for adjusting sex ratios and functional haplodiploidy has evolved several times independently in species with LMC, presumably to allow them control of their sex ratios despite inbreeding [13]. It also allows the production of accurately skewed sex ratios [15].

In haplodiploid taxa, parental inbreeding causes daughters to be more related to their mothers, while son-to-mother relatedness is unaffected, which further skews the selected sex ratios towards females [16, 17]. Hamilton [16] showed that the evolutionarily stable strategy (ESS) sex ratio (fraction of sons) when all patches are colonised by *n* mothers (called foundresses) all laying identical clutches is (see Additional file 1: text and figures and [18]):

1 $$r* = \frac{n - 1}{n} \cdot \frac{1}{2} \cdot \frac{1 + F}{{1 + 2F}} = \frac{n - 1}{n} \cdot \frac{1}{2} \cdot \frac{4 - 2s}{{4 - s}} = \frac{n - 1}{n} \cdot \frac{1}{2} \cdot \frac{4n - 2}{{4n - 1}}$$

with *F* = inbreeding coefficient and *s* = probability that a female is sibmated.

Equation (1) was not meant to be used when *n* varies [18]. When foundress number varies (and mothers produce different sex ratios and clutch sizes) then the rate of sibmating changes as foundress numbers change and this needs to be taken into account [17, 18]. It results in more frequent sibmating that in turn results in higher *F*, which results in a more female-biased ratio than Hamilton calculated. Frank [18, 19] and Herre [17] accounted for this and obtained bulky equations that require many parameters to be estimated. Herre [17] simplified his bulky solution by assuming that all patches produce equal numbers of emerging females, whatever the number of foundresses. This may happen when, as is observed, (i) the sex ratio increases with foundress number and ii) the foundresses have reduced fecundity when foundress numbers increase. Herre’s [17] assumption allowed him to estimate the sibmating rate as the reciprocal of the harmonic mean foundress number (1/*n*_h_) giving an *F* = 1/(4*n*_h_—3) and Herre [17] calculated the ESS sex ratio in a patch containing *n* mothers as:

2 $$r_{n} * = \frac{n - 1}{n} \cdot \frac{1}{2} \cdot \frac{1 + F}{{1 + 2F}} = \frac{n - 1}{n} \cdot \frac{1}{2} \cdot \frac{{4n_{h} - 2}}{{4n_{h} - 1}}$$

Herre’s [17] estimate of sibmating is slightly higher than Frank’s which was based on observed wasp production, given specific foundress numbers for his study species [18, 19].

Similar to Eq. (1), the last fraction takes into account the relative genetic value of males and females. In diploids where males and females have equal value the last term is 1 and in haplodiploids it is equal to (1 + *F*)/(1 + 2*F*) [20]. *F* can be estimated from foundress numbers (*n*_h_), or it can be estimated more accurately from population genetic data. In fig wasps [16, 21–27], but also more generally, the degree of sibmating, and hence *F* varies over time and space and depends on foundress numbers of previous generations. It is unrealistic to expect foundresses to have information about the current *F* and it seems reasonable that sex ratios evolve in line with the mean *F* [20].

**Fig. 1:**
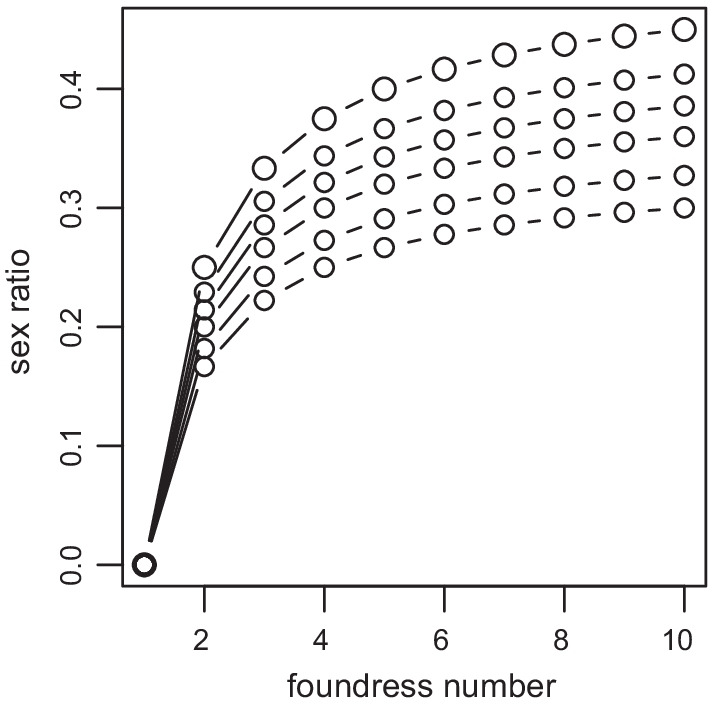
The ESS sex ratio (fraction of males) under LMC. The sex ratios predicted by Eq. (2) for values of *n* when *n*_h_ respectively = ∞, 3.25, 2, 1.5, 1.16 and 1 from top to bottom. These values correspond to values of *F* = 0, 0.1, 0.2, 0.3, 0.6 and 1. Lines connect points with the same *n*_h_

Considering different values of *n* and *n*_h_ (or *F*), Eq. (2) shows the three main predictions of LMC theory (Fig. 1). (1) The predicted sex ratios are female biased when there are few foundresses. (2) The skew is more intense at lower foundress numbers and approaches equality as the number of foundresses increases (i.e. the patch is more like a panmictic population). (3) Higher values of *F* (lower *n*_h_) as would be expected for more inbred species, result in more female biased ESS sex ratios. Notice how the effect of increasing *n*_h_ on the ESS sex ratio rapidly decreases as *n*_h_ increases (a change from 1.16 to 1.5 almost has the same effect on ESS sex ratios as an increase from 3.25 to ∞).

Another important aspect of Fig. 1 is that although Eq. (2) predicts that the sex ratio for 1 foundress should be zero, Hamilton already pointed out in 1967 that the model assumes that all females will be mated [13]. Therefore, the theory predicts that a single foundress should lay just enough sons to ensure that all her daughters will be mated [13]. Nagelkerke [28] extended LMC theory to take finite clutch sizes into account and his model predicts sex ratios for single foundresses. However, when clutches are larger than 40, as is true for fig wasps (mean clutch size of 39 species = 182 and minimum = 51, see Additional File 4), Nagelkerke's predictions drop below the commonly observed sex ratio of 0.1 for single foundress fig wasps (mean for 39 species = 0.12, see Additional File 4). This is presumably because the required number of males per female is higher than Nagelkerke's model assumption [28]. Therefore, current models do not make an explicit prediction for single foundresses that can be tested. As a result we will use 0.1 to look at the fitness effect of adjusting sex ratios in the calculations below.

When females do not lay equal clutch sizes, several optimum predictions can be made, depending on the information females have. First, in haplodiploids, *F* estimated using the harmonic mean, *n*_h_, is too low [18]. Therefore, haplodiploids' sex ratios with unequal clutches should be more female-biased than when clutches have equal sizes. To avoid confusion we use clutch to refer to the eggs of a single female and brood to refer to all the eggs laid in a patch. Second, when females do not know if they lay the larger or the smaller clutch, the brood sex ratio will be more female biased than Eq. (2)'s predictions [18]. Third, if females know their own relative clutch size, Yamaguchi [29] showed that in diploids there is a threshold number of offspring that should be sons and all offspring in excess of this threshold number should be daughters. Kjellberg [30] confirmed this for haplodiploids. Hence, when mothers have knowledge of their relative clutch sizes, then optimality models predict broods (combined clutches in a patch) that have a sex ratio equal to, or slightly below Eq. (2). Note however that smaller clutches will have more sons (or even only sons) and larger clutches will have more female biased ratios than Eq. (2) predicts.

Nunney and Luck [20] showed that when females do not know the foundress numbers for their patches, they should adjust their sex ratio to the average foundress number.

While the sex ratio predictions of Fig. 1 are well known, the fitness consequences of adjusting sex ratios are not (but see [11, 28]). The fitness consequences depend on the strategies other females are using. This can be understood by the following example: in a panmictic population, having sons will have high fitness returns if other females produce mostly daughters, but will be terrible if all females are producing mostly sons. This is the frequency-dependent nature of fitness for sex ratios that Düsing understood for panmictic populations [31, 32] and which Hamilton developed for the situation of LMC [13]. Sex-ratio fitness-landscapes are thus ever-changing with the landscape changing as trait values change.

**Fig. 2:**
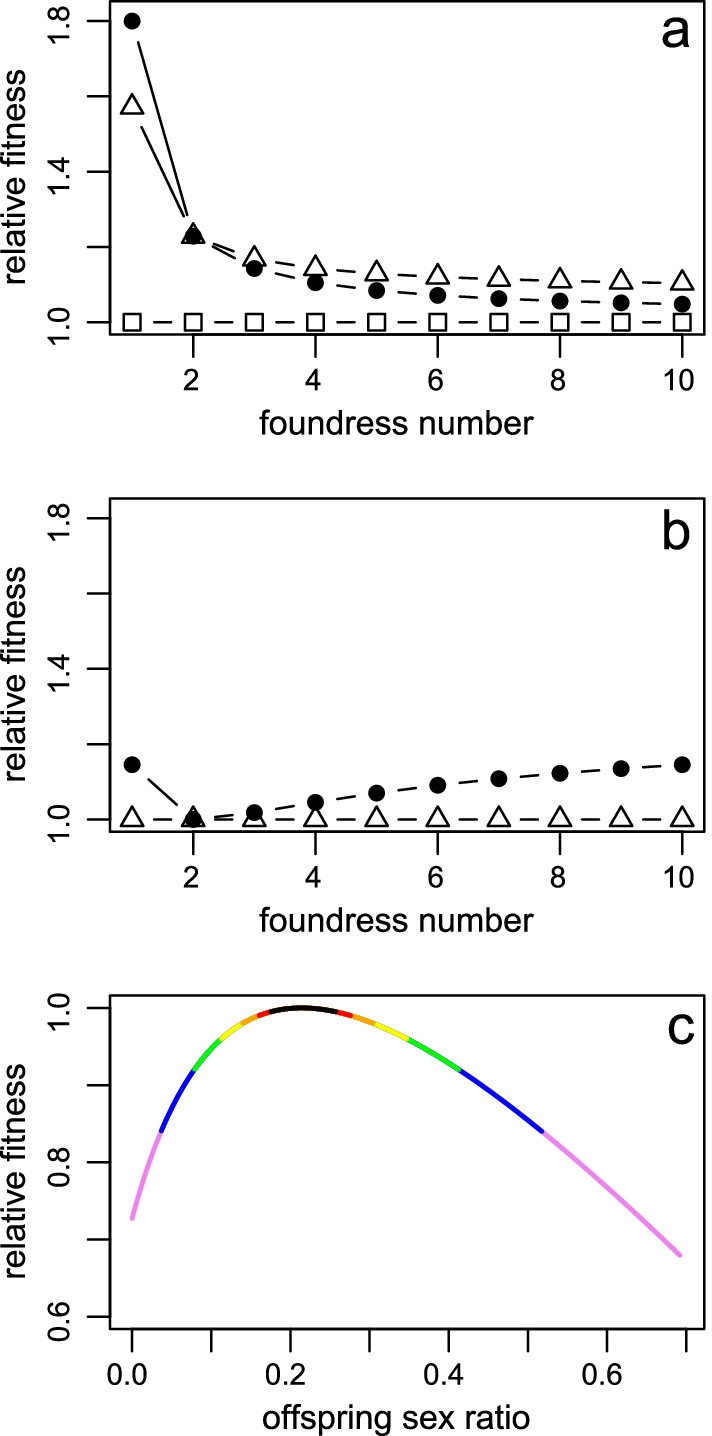
Fitness consequences of biased sex ratios. Relative fitness compared to (**a**) an unadjusted sex ratio of 0.5 and to (**b**) a constant ratio of 3/14 of a focal foundress when she lays an unadjusted sex ratio of 0.5 (open squares; UA females), a constant, but adjusted sex ratio of 3/14 (open triangles; IA females), and an adjustment for foundress number as predicted in Eq. (2) and 0.1 for single foundresses (solid circles; E2 females). Lines connect relative fitnesses of each kind of focal female. **c** The relative fitness of a range of sex ratios compared to the optimal ratio of 3/14 in a two-foundress patch where the other female lay the optimal ratio of 3/14. Colours indicate relative fitness brackets: black is *w* > 0.995, red is 0.995 > *w* ≥ 0.99, orange is 0.99 > w ≥ 0.98, yellow 0.98 > w ≥ 0.96, green is 0.96 > w ≥ 0.92, blue is 0.92 > w ≥ 0.84, violet is 0.84 > w ≥ 0.68. The fitnesses are calculated for an *n*_h_ = 2 using equation (S1)

In the following we simplify matters by just looking at three extreme sex ratio strategies; females that (1) produce an unadjusted 50:50 sex ratio (UA females), (2) produce an invariant, but adjusted sex ratio of 3/14 (IA females, the optimum for n = 2 in Eq. (2)) and (3) produce Eq. (2) sex ratios (E2 females). Consider a population with a constant inbreeding coefficient, *F* = 0.2 (*n*_h_ = 2). The relative fitness of a focal female that plays against a population that is composed of UA females is given in Fig. 2a, and the relative fitness when playing against IA females is given in Fig. 2b. Under LMC conditions, females that bias their offspring sex ratio towards females increase their own fitness (Fig. 2a). Especially for one foundress patches this increase is the highest and will be higher the more biased the sex ratio is (provided there are enough males to mate with all their sisters). In this example when the sex ratio is biased to 10% males, the increase in fitness is a massive 80%. Even just biasing to 3/14 (≈ 20% males) results in a 57% fitness increase. The fitness increase is much less marked for higher foundress numbers, dropping sharply to 23% for two foundresses. Interestingly, E2 females would do less well than IA females at higher foundress numbers, when playing against UA females. The switch over of fitness between E2 and IA females depends on *n*_h_ and the sex ratio of the IA females.

However, the fitness landscape changes as the population evolves over it. When the population evolves to be more female biased (Fig. 2b; IA would actually never be able to fix) the E2 females will start to do better than IA females and their frequency will increase. The benefit of adjusting sex ratios to foundress numbers, although substantial, remains modest in comparison to the fitness benefit of producing biased sex ratios as opposed to unbiased ratios. Since *n*_h_ = 2 in this example, we should not see high foundress numbers frequently. Therefore, the benefits of accurate adjustments at foundress numbers far from *n*_h_ will be infrequent and the fitness effects will be limited. These calculations show that having a biased sex ratio in a 50:50 environment gives a much larger fitness advantage than a variable and biased sex ratio in a biased sex ratio environment. Nunney and Luck's [20] model where mothers bias their ratios to the average situation gives mothers the large benefit associated with female biased ratios, while the marginal benefits of facultative adjustments are not reaped.

The fitness penalties of sex ratio strategies increase as the strategies are further away from the ESS sex ratio (Fig. 2c). The ESS sex ratio for two-foundresses is 3/14 when *F* = 0.2. Fitness consequences of imperfect sex ratio adjustment when in competition with other females is highest when the fitness curve is the steepest on both sides of the optimum, i.e. for two foundress figs (calculus not shown). We consider the fitness of a focal female when the non-focal female lays the ESS strategy of 3/14 using equation (S1). There is a range of 0.011 around the ESS that will have a relative fitness of 0.9999 or larger. That is equivalent to a selection coefficient of 0.0001 or smaller. Since fig wasp populations are substantially larger than 5000 individuals [33], selection should be effective at removing strategies with a relative fitness of 0.9999. If genotypes map simply to phenotypes, phenotypes map simply to fitness (i.e. no pleiotropy) and the environment remains constant, we can expect sex ratios to be very close to predicted optima. However, natural conditions are variable with variation in other females' expected offspring number, own offspring number, sex ratios, *F* and seasonal fluctuations in abundance [25]. An example illustrates the dilemma best. Consider the above case when mean clutch size is 210. The ESS sex ratio for two females is 45/210 (= 3/14). If the non-focal mother lays exactly 45/210 then the optimal strategy is 45 sons and 42—48 sons (the remainder being female) will give a relative fitness of 0.999. Now consider a situation where the non-focal female laid 10% more sons (50) and laid 10% less eggs in total 199. Now the optimal number of sons is 44 out of 210 and the 0.999 range shifted to 40—47 sons. Similarly, if the focal female lay 10% more eggs then the optimal number of sons is 46 and the 0.999 range is 42—50. In this way, a variable environment will, if mainly sons are laid first followed by mainly daughters (slope strategy, see below), result in an inability to fix a single optimal phenotype. We therefore expect that wasp sex ratios should vary and a theoretical optimum needs to consider the environmental noise.

### The fig wasp model system

Here we present pollinating fig wasp traits that are in line with model assumptions and for the moment, we refrain from pointing out deviations. However, with 1000 + pollinating wasp species, variation is expected.

**Fig. 3:**
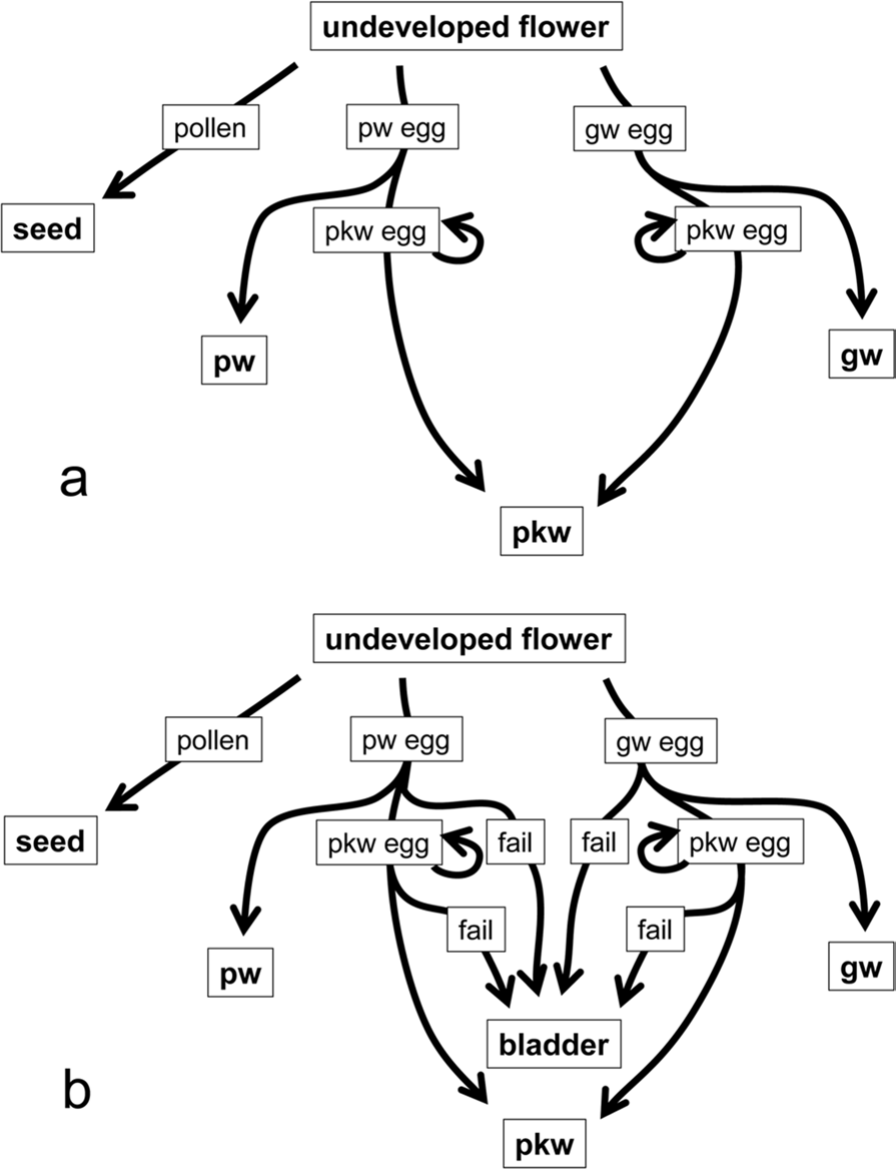
The development of the female flowers of *Ficus*. **a** Consider the existence of bladders to be unimportant. **b** Considers the existence of bladders. They will develop into seeds, galls containing pollinating wasps (pw), gall wasps (gw) or parasitoid and kleptoparasitoid wasps (pkw) or bladders. Bladders are the result of developmental failure

Pollinating fig wasps conform well to the local mating requirements. In brief, their life cycle is as follows (summarised from Kjellberg et al. [34]). Male pollinating wasps chew an exit hole out of the fig. Pollen carrying mated female wasps leave through the exit hole and search for receptive figs with the help of wind and chemical attractants. One or a few female wasps enter the fig that contain the enclosed receptive female flowers by crawling/squeezing through the ostiole, a tiny, bract-lined opening at the apex of the fig. In doing so, they become foundresses, the mothers of the next generation. They frequently lose their wings and part of their antennae break off while passing through the ostiole. Once inside they do two things, (1) pollinate flowers actively or passively and (2) lay their eggs. The inside of the fig is lined with many uniovulate pistillate flowers. The course of the flower’s development depends on what happens next and here we describe the original idea that acknowledged the potential influence of mortality on sex ratios, but dismissed it as unimportant (Fig. 3a). If nothing else happens and the flower is not pollinated it will remain an unpollinated flower; if it is pollinated, it will develop into a seed if no wasp egg is laid in it. The fates of the flowers (pollinated or not) depend on the oviposition behaviour of four wasp guilds. These are the pollinating wasps, ovule gallers, parasitoids and kleptoparasites. Kleptoparasites have essentially the same effect as parasitoids and will be combined with the parasitoids in the following. Seed predators do occur [35], but these are infrequent and we ignore them here. Pollinating wasp foundresses lay a single egg per flower [36, 37] which will either develop into a male or a female pollinating wasp. Ovule galling wasps will gall the uniovulate flowers (without pollinating wasp eggs or before oviposition by pollinating wasps) and parasitoids parasitize pollinating wasps, gallers and other parasitoids. Early gallers can sometimes gall the fig wall instead of flowers. The wasp and fig development is synchronized so that the wasps become adults and male flower anthesis occur at the same time. Male pollinating wasps chew themselves out of their own galls and search for galls containing females; they chew a mating hole into the gall and mate with the females who will frequently be their sister. Once the female wasps emerge from their galls they collect pollen actively or passively and leave the fig through the exit hole cut by the males or sometimes the ostiole, to start a new cycle.

Overlaying the basic life history are a number of other features that make fig wasps ideal LMC candidates. Adult females have a very short lifetime [27, 38] and once figs are pollinated they rapidly lose attractivity to pollinating wasps [39, 40] so that wasps will oviposit more or less at the same time. Matings are restricted to a female wasp’s natal fig. Since fig wasps are haplodiploid, fertilized eggs develop into females while unfertilized eggs develop into males.

Foundresses interfere with each other during oviposition so that they lay fewer eggs in the presence of another than when they are on their own [41–44]. But when they oviposit sequentially more eggs develop per foundress as long as the fig has sufficient flowers to oviposit into, showing that interference is during oviposition and not during development [41, 44]. This is because each wasp larva is sequestered in an individual flower and because more developing wasps seems to create a more effective resource sink [45, 46]. This is an important difference with parasitoids that compete for shared finite resources in a parasitized larvae, so that wasp development is poorer the more eggs are laid in one host. However, there is not much data to support or refute the claim that there is no competition between fig wasp larvae developing in a fig. One data set that allow us to test this idea indirectly shows that the number of wasp offspring developing in a fig affects neither the mean wasp size of developing daughters (linear model: *P* = 0.217, see Additional file 1) nor the standard deviation of their size (linear model: *P* = 0.189, see Additional file 1).

It is assumed in the basic models that foundresses' clutches have similar sizes [7, 18]. However, different sizes can be incorporated in the theory. In order to calculate the rate of sibmating from foundress numbers, it is standard to assume that mating is random within a fig. Random mating is also necessary to calculate the mating prospects of males (see Additional file 1) and this seems to be a reasonable assumption [7].

In addition they possess traits that allow testing of the theory—foundress numbers vary in nature, foundresses die inside figs so that *n* can be determined from naturally populated figs [16, 17, 47–50]. The harmonic mean foundress number can be calculated from the corpses of dead foundresses. Species have a marked sexual dimorphism [51] which allows males and females to be counted accurately. Females are proovogenic meaning all their eggs are mature as soon as they leave their natal fig [52]. Females readily enter the fig and oviposit if they are placed on a receptive fig allowing experimental manipulation of foundress numbers [18, 19, 26, 41, 53–70]. Females do not invest in offspring rearing and no egg dimorphism is known [52], meaning the investment in the two sexes can be equated with the number of eggs of each sex that were laid. Wasps are sustained by the gall that receives nutrients from the plant [71]. Since wasps develop in the confines of the fig, and because figs are selected to breed wasps, it was natural to assume that their mortality must be low. This would mean that the sex ratio of the laid eggs, or primary sex ratio, which is the trait predicted by models, should be very close to that observed among emerging adults, the secondary sex ratio.

For pollinating fig wasps, a single foundress should lay enough sons to ensure all her daughters will be mated, but in addition, there must also be enough males to chew an exit hole through the fig wall [16]. Several studies have found that all females are not always mated [49, 50, 68, 70, 72–75] and sometimes too few males are laid to release their sisters [48, 76]. While a single *Kradibia tentacularis* male is sufficient 63% of the time, more males are more successful [76].

**Fig. 4:**
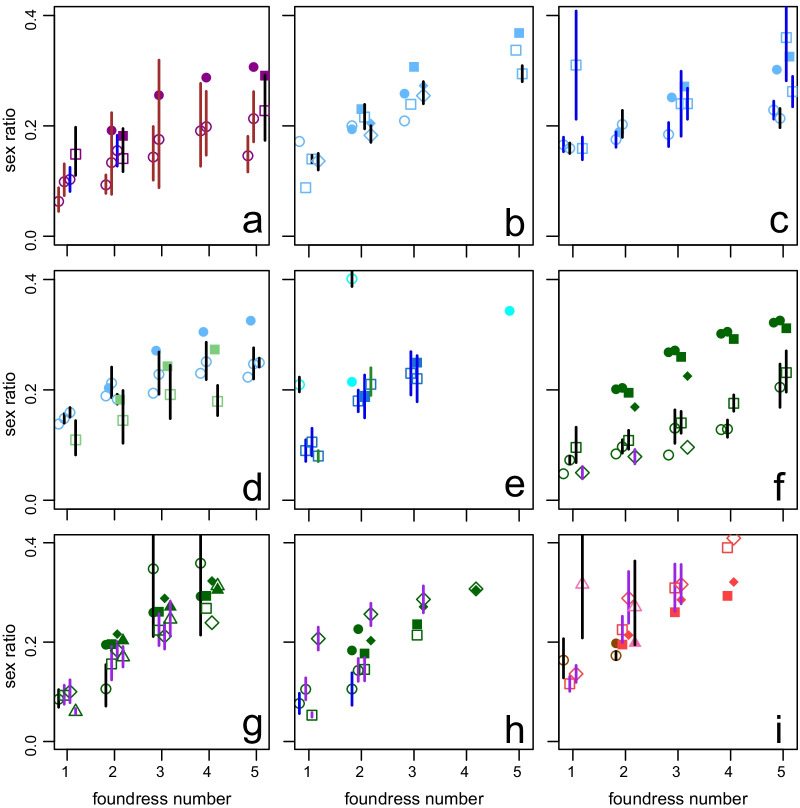
Sex ratios increase with foundress numbers, but not enough. Observed (open shapes) and expected (solid shapes) sex ratio variation in 36 data sets from 25 fig wasp species. Within each graph, each shape refers to a different species. We obtained 95% CIs in five ways indicated by the colours of the vertical lines and in order of preference as data allowed: (1) In black, e^a^/(e^a^ + 1) where a is given by the generalized linear model coefficient ± 1.96x(se of the coefficient). A model was fitted for each foundress number and a quasibinomial statistical model was used if data was overdispersed. (2) In brown e^a^/(1 + e^a^) where a is the mean of log(p/(1-p)) ± 1.96x(se of the mean). (3) In purple, (sin(a))^2^ where a is the mean ± 1.96x(se) of arcsine transformed data. (4) In blue, mean sex ratio ± 1.96x(se). (5) In green, given. **a** *Blastophaga nipponica* (circles) and *B. psenes* (squares), **b** *Ceratosolen capensis* (circles), *C. fusciceps* (squares) and *C. galili* (rombi), **c** *C. gravelyi* (circles) and *C. marchali* (squares), **d** *C. solmsi* (circles) and *Elisabethiella baijnathi* (squares), **e** *Eupristina koningsbergeri* (circles, mean sex ratio and 95% CI of 5 foundresses is not plotted and is 0.46–0.47–0.49), *Kradibia tentacularis* (squares), **f** *Pegoscapus aerumnosus* (circles), *P. franki* (squares) and *P herrei* (rombi), **g** *P. jimenezi* (circles), *P. longiceps* (squares), *P. lopesi* (rombi), and *P. piceipes (triangles)*, **h** *P. silvestrii* (circle), *P. tonduzi* (squares) and *Pegoscapus* sp*.* ex *F. crocata* (rombi), **i** *Platyscapa awekei* (circles), *Tetrapus ecuadoranus* (squares), *Tetrapus* sp. ex *F. insipida* (rombi) and *Valisia javana* (triangles). Expected values were calculated using Eq. (2). Details in Additional files 1 and 2 Supplementary text and figures. Genera are colour coded as in later figures

In general, fig wasp sex ratio behaviour seems to fit the qualitative LMC predictions [2]. They produce female biased sex ratios that become less female biased as foundress number increases (Fig. 4 summarises data from 36 data sets on 25 wasp species [7, 16–19, 26, 27, 41, 49, 50, 53, 54, 56, 57, 59–62, 64, 66, 68, 70, 77, 78]). In only two of 33 data sets with data for two-foundress figs, and one of 25 data sets with data for three-foundress figs did the proportion of males decrease as foundress number increased. Some evidence suggests that more inbred species, *i.e.* low mean foundress number species, lay more female biased sex ratios [7, 17, 53]. In five species from three genera the number of sons laid per foundress increases as foundress number increases [19, 57, 68, 70, 79]. In two species, *Elisabethiella baijnathi* and *Blastophaga nipponica*, the creation of a social environment of two or more foundresses resulted in an increase of the number of sons even though only one foundress was allowed to produce offspring [53, 57]. Finally, it has been argued that deviations from optimality is greater in scenarios that are encountered less frequently [47, 80, 81]. In fact, pollinating fig wasps seem to be so well adapted that they have been called “wonderful” [7] and West et al. [2] endorsed the good fit. Greeff and Newman [66] even found that females seem to take a co-foundress’s clutch size into account. In a review of 23 fig wasp species Herre et al. [7] concluded that:

“Nonetheless, in the face of the accumulating support, it is either an ironic twist, or poetic justice, that the fig-wasp data conforming least well to Hamilton's predictions of facultative sex-ratio adjustment appear to be those collected by Hamilton himself.”

## Approaches to study fig wasp sex ratios

Several approaches have been used to investigate fig wasp sex ratios and these can introduce systematic biases. Observational studies are where dead foundresses are counted just before the next generation chews their way out of their galls [16, 17, 47–50]. With this technique figs do not need to be enclosed in mesh bags prior to receptivity nor is it required to enter specific numbers of wasps into figs. While this approach has the benefit that biologically realistic conditions prevailed during oviposition and development it has some drawbacks. (1) It is not known why different numbers of females entered a fig. Under natural conditions foundress number may covary with other factors that may also affect the sex ratio such as the size of the fig and how long the fig waited to be pollinated [69, 82]. (2) It is not known if the females entered at roughly the same time [58] which is problematic because models give different predictions for sequential versus simultaneous entry (see below) [54, 83, 84]. (3) Foundresses of many species leave figs after oviposition and the foundress number cannot be inferred accurately [85–88]. This will result in a systematic underestimate of the foundress number and sex ratios that appear to be too high (the opposite is observed, see below). (4) Since ovule gallers, parasitoids and kleptoparasites are not prevented from ovipositing in the figs, these wasps will influence realized clutch sizes and may change the secondary sex ratio [89, 90]. The latter occurs because females are believed to be more exposed to parasitism [89, 91]. We discuss this issue in more detail below. (5) Interactions between foundresses can have an effect on clutch size and hence sex ratio. We discuss this below as a potential explanation for too female-biased sex ratios.

The alternative to using naturally founded figs is to enter wasps experimentally into figs that are shielded from oviposition by other wasps by tying mesh bags around the figs [18, 19, 26, 41, 53–70]. In this way a proper experiment can be performed with knowledge that wasps entered at more or less the same time (avoiding sequential interpretations [19]) and that figs entered by different numbers of wasps are equivalent. This approach is not devoid of problems, (1) keeping wasps together in polytops or bags upon exiting their natal fig and before entering the next, and the lack of active dispersal, may signal or remove important proximate cues mothers use to adjust their sex ratios. (2) The mesh bags will affect fig photosynthesis and hence to some extent nutrient availability that should affect wasp mortality. (3) Interactions between wasps can affect the outcome of experimental studies as well and we discuss it as a situation that can result in too female-biased sex ratios.

Two methods have been used to prevent females from ovipositing successfully [92]. Both of these have been used to create a situation where a fig contains several wasps but only one can oviposit [53, 57]. In the first, females’ ovipositors are sliced off which prevent them from ovipositing [53]. Although the social cue of another wasp is present, it may not present potential cues such as probing time to find suitable ovules to oviposit in and there is no guarantee that cut individuals behave normally. In the second, females are irradiated so that although they lay eggs, these eggs do not develop [57].

Normally the two methods of obtaining figs that vary in foundress number are combined with counting of the offspring. More recently, genotyping with microsatellites have been used to determine the number of foundresses [75, 93], number of fathers [63] or to discern the clutches in broods [60, 66]. However, when only a sample of offspring are genotyped individual female’s clutches cannot be reconstructed. Molecular techniques also make it possible to estimate *F* directly [66, 93–95].

Other methods for studying sex ratios are also employed. By killing foundresses at different time intervals after entry, the sequence in which the sexes are oviposited has been determined. Oviposition has been interrupted by killing foundresses inside the fig through dipping figs in hot water [68], or by injecting ether [41, 65] or poison [64] into the lumen of the fig via the ostiole.

Due to the haplodiploid sex determination, females that are prevented from mating by removing galls without mating holes, will lay male eggs only [92, 96]. This is a useful tool to study sex specific mortality when pollination can be ensured [96].

Zhang et al. [97] introduced another technique. They removed *Ceratosolen fusciceps* foundresses from figs they entered and let them enter new figs in a species where females do not naturally re-enter figs. In doing so they illustrated that *K. tentacularis*’s resetting to laying mostly males first upon entering another fig [64] is an exaptation (sensu [98]) rather than an adaptation to a new situation (also see [88]).

## Exact and binomial primary sex ratios, mortality and secondary sex ratios

**Fig. 5:**
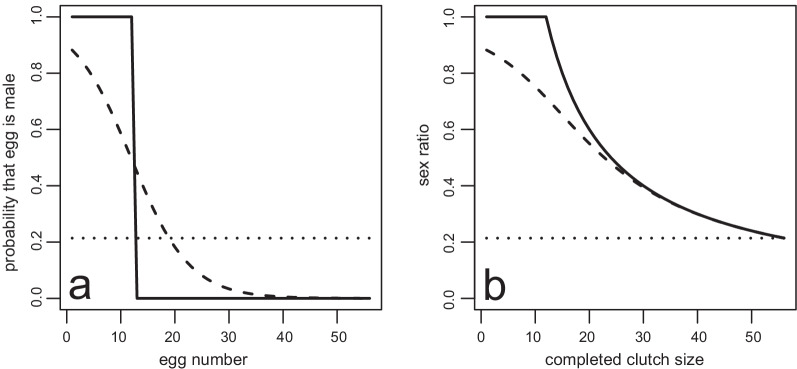
Three modes of sex ratio precision and the resultant sex ratio-clutch size relationship. **a** The probability that successive eggs will be male and **b** the sex ratio at a certain completed/terminated clutch size for the three sex ratio strategies: exact (solid line), slope (dashed line) and binomial (dotted line) for a clutch of 56 that all result in a mean completed sex ratio of 3/14 after 56 eggs were lain. For the slope strategy the probability that egg number x will be male is given by *c*e^*a*x^/(*c*e^*a*x^ + 1-*c*), with *c* = 0.9 and *a* = -0.1849463

Models typically assume exact sex ratios while a classical null expectation for sex ratios is that they are binomially distributed [99, 100] although Green [15] illustrated the benefit of more accurate sex ratios in haplodiploids. A reduction in sex ratio variance will increase fitness [15, 28, 80] but will not affect sex ratio predictions much [101]. The binomial expectation is easy to understand as a scenario where all eggs have the same probability of being male (Fig. 5a), say *x* here. An exact sex ratio requires that a specific fraction *x*, of eggs must have a probability of one that they will become male and the remaining eggs a probability of zero that they will be male. If sex ratios are to be more precise than binomial and less precise than exact, an intermediate situation must occur where some eggs are more likely to be male than *x* and the remaining eggs less likely to be male than *x*. In fig wasps, females typically lay most male eggs first and then lay mostly female eggs [41, 64, 65, 68]. In other words, the probability of an egg being male is a function with a negative slope (Fig. 5a). We will refer to this intermediate accuracy strategy as “slope”. Figure 5a combines these precision arguments with what we know about fig wasps to show what an exact, binomial and slope strategy would look like (Fig. 5). The slope strategy indicated in Fig. 5a was obtained with a reverse S-shaped function [102] and parameterized so that the final sex ratio would be 3/14 (Fig. 5b). The choice of a function is not limited to this one. Note that greater precision does not mean males must be laid first, the same can be achieved by laying females first. However, if own clutch size is not known beforehand, as is probably the case, it would be safer to start with mostly males (compare to [103]). This sequence was selected to match fig wasp behaviour which is similar to the slope strategy (Fig. 5; [41, 64, 65, 68]).

Waage [104] suggested that specific sequences of fertilized and unfertilized eggs can increase precision of sex ratios and non-random sequences have been found in several wasps other than fig wasps [103, 105, 106]. Waage and Ng Sook Ming [103] also argued that a specific sequence could result in the desired biased sex ratio. Some have argued that the sequence is a result of the rate of oviposition [105, 107, 108]. The proximal explanation that involves an inability to sustain the release of sperm from the spermatheca [106] agrees with a slope strategy where most males are oviposited first when eggs are laid faster [64]. However, it conflicts with observations that interference, which should slow down oviposition rate, increases sex ratios. These differences suggest that a more subtle mechanism is at work. Even so, observed switches in the sequence of fertilized and unfertilized eggs in parasitized and unparasitized hosts of other wasps hint that our suggestion of a switch in a different environment (see below) is not unrealistic [109].

**Fig. 6:**
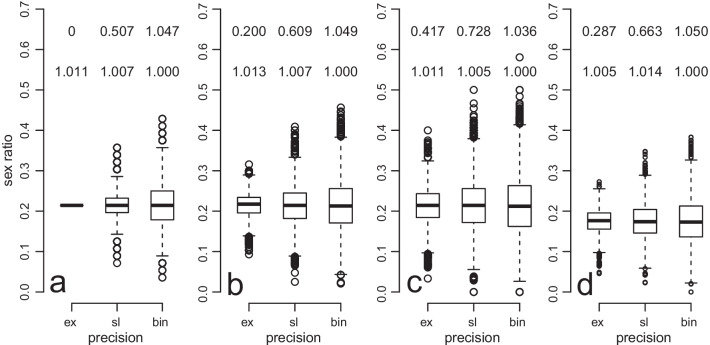
Sex ratio variation in individual clutches and fitness consequences in two-foundress figs of three levels of precision. Sex ratio variation for 10,000 pairs of each precision type for **a** a primary sex ratio of 3/14, **b** constant 20% per egg mortality, **c** constant 40% per egg mortality and (**d**) sex-specific per egg mortality of 30% for male eggs and 10% for female eggs. These are standard box and whisker plots with the heavy line indicating the median sex ratio. To calculate fitness each fig consisted of a focal female of the stated precision and a binomial non-focal female. The mean fitness of 10,000 focal females were calculated using equation (S1) for when *n*_h_ = 2. These were converted to relative values by dividing by the fitness of binomial focal female's which is the bottom of two values given above each box. The heterogeneity factor (hf) is also given above the relative fitness for each plot. Each estimate is based on 10,000 clutches that were 56 eggs in size before mortality and were generated in R. No male-less broods occurred

The observed variance relative to the expected binomial variance can be expressed as the heterogeneity factor (hf) and is equal to 1 if the variance is binomial, less than binomial if hf < 1, and greater than binomial variance when hf > 1. The greater variance of binomial over slope and slope over exact can be seen in Fig. 6a. In two-foundress figs, higher accuracy leads to a small fitness increase of just over 1%.

Another phenomenon that can increase variance in sex ratios is mortality. Initially it was assumed implicitly that mortality in fig wasps is low meaning that the observed sex ratios of adult offspring after mortality (secondary sex ratios) are approximately equal to that of the primary sex ratios. However, pollinating fig wasps can suffer severe and variable mortality [100]. These can be estimated by looking at the number of pollinating wasp eggs and offspring, the number of galls, the number of bladders (empty galls) and the number of parasitoid fig wasps in a fig (Fig. 3b). Each wasp stems from a gall in which it develops and which can be identified as a hollow oval structure with a little hole the wasp chewed to escape. Some flowers turn into similar hollow oval structures without holes and these are known as bladders. A direct observation of fig content just after oviposition showed that *K. tentacularis* oviposits in about 95% of the flowers, while at wasp emergence, about 40–50% of the flowers contain a wasp and 30–40% of the flowers have turned into bladders [37]. Similarly comparing data on numbers of emerging offspring and numbers of bladders in pollinated and unpollinated figs [37, 110] and parasitized and unparasitised figs [90] shows that each bladder represents an egg that did not develop into an adult wasp. The number of bladders suggest high mortality rates (*C. fusciceps*: 27% [90]; *C. marchali*: 16–50% [111]; *Valisia javana*: 26–28% [112]; *K. tentacularis*: 30–52% [37, 110]; *Eupristina altissima*: 24–57% [113]; *Eupristina belagaumensis*: 7–25% [58]; *Tetrapus costaricana*: 4.2% [45]; *T. americanus*: 0.5% [45]; *Pegoscapus tonduzi*: 24% [45]; *P. piceipes*: 12–19% [45]; *P. hoffmeyeri* 1 & 2: 13–20% [45]; *P. gemellus* 1 & 2: 1–12% [45]; *E. baijnathi* 13–29% [114]). Mortality varies with foundress number and if flowers are pollinated or not [71] but these trends vary across taxa.

Mortality is thus common and sizable in pollinating fig wasps. While the effect of an increased variance can clearly be seen from the box and whisker plots after 20% and 40% per egg mortality rates (Figs. 6b and 6c), the corresponding hfs do not increase much beyond 1 (in fact it is smaller in one case). In the simulated two-foundress figs more accurate sex ratios has higher fitness than a binomial strategy, but only by just over 1% (Figs. 6b and 6c). When mortality is independent of sex [71] it will increase sex ratio variance without skewing the sex ratio (Figs. 6b and 6c).

If, on the other hand, mortality is dependent on the sex of the egg and varies among figs, then the secondary sex ratio can be skewed away from the optimum in addition to the variance being enlarged (Fig. 6d). Li et al. [96] suggested that developmental mortality is much more for male (23%) than female larvae (less than 7%) in *C. fusciceps*. On the other hand, Galil and Eisikowitch [92] found that female *C. arabicus* are more prone to developmental mortality. Since LMC theory predicts the primary sex ratio, such deviations will not alter the optimal investment, it will simply appear, from secondary sex ratios, as though the sex ratios are not optimal.

A second cause of mortality that can sometimes be sex dependent is due to parasitoids. Since fig wasps typically lay their female eggs closer to the outside of figs where exposure to parasitic attacks is higher it is expected that it should bias sex ratios to males [89, 91]. This should be especially marked in figs with higher foundress numbers when more pollinating females are laid closer to the surface of the fig [91, 115]. In seven species where parasitoids reduced the number of pollinating wasps, sex ratios were increased in two [67, 77, 116], the results were ambiguous in one [117, 118] and there was no effect on sex ratio in three [27, 61, 90]. In one species the number of females was reduced more than the number of males [119]. Kleptoparasites may have a similar effect [120]. An important caveat with such correlational studies is that parasitoids/kleptoparasites attack figs with more wasp progeny more frequently. This higher attack on higher foundress figs can lead to spurious positive correlations between non-pollinating fig wasp numbers and sex ratios [117]. Therefore, parasitoid effect studies are only accurate if foundress number and parasitism was controlled [117]. Although it is thought that mortality due to parasitoids should come at a cost of one pollinating wasp per one parasitoid this exchange rate can be as high as 1.8 [90], probably due to probing.

**Fig. 7:**
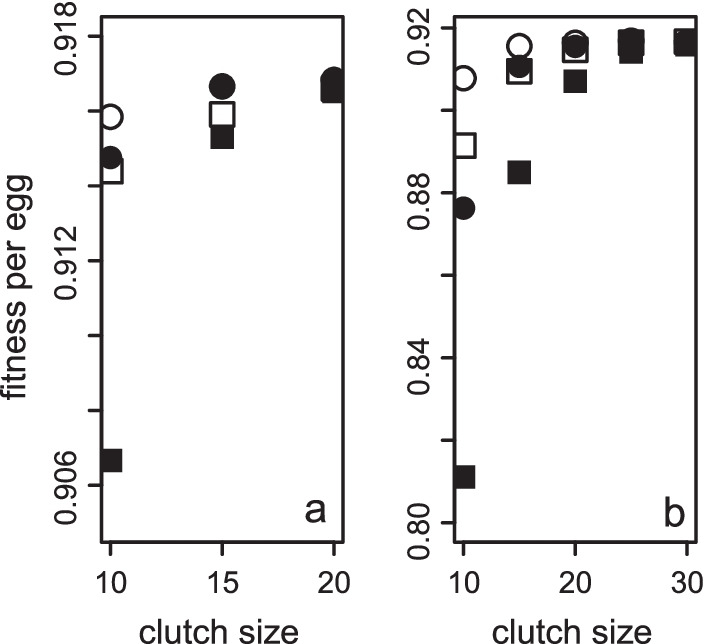
The fitness effect of variance on small clutches. The per egg fitness of a focal female adopting the slope (circles) or binomial (squares) strategy in **a** two-foundress and **b** one-foundress patches and if all (solid) or only broods with more than zero males (open) are considered. For the slope strategy the probability of an egg being male was calculated as in Fig. 5 with *c* = 0.9 and* a* calculated to give a final sex ratio of 3/14 for the completed clutch. It was parameterized to the 4^th^ decimal. Fitness was calculated using equation (S1) with *n*_h_ = 2 for a mean of 100 000 generated at each of several clutch sizes

The variance of sex ratios increases with probabilistic strategies as clutches get smaller. Therefore, the loss in fitness due to probabilistic strategies is greater when clutches are smaller (Fig. 7), but not very much (Fig. S1). The effect is less severe the more precise the strategy is (compare squares to circles in Fig. 7) and the larger the clutch is (compare same symbols for different clutch sizes in Fig. 7). The biggest fitness cost is due to maleless patches (compare open to filled symbols in Fig. 7). However, when the clutch size exceeds 20, penalties due to maleless patches become negligible (Fig. 7). While average clutch sizes are normally larger than 20 (mean clutch size of 39 species = 158 and minimum = 51, see Additional File 4), variation between foundresses sharing a fig can be substantial [58, 60] and should result in clutches that are smaller than 20.

Fig wasp sex ratio data are usually overdispersed [49, 66] compared to the binomial distribution. While mortality did not increase the hf much beyond 1 this situation should be different when mortality rates are sex dependent and vary among figs. Additionally, the high overdispersion of observed data suggests that foundresses of species are not monomorphic for their sex ratio strategies and/or that we have not included important parameters in our statistical models. In the light of a slope strategy, clutch size is an important variable that must be included [100]. The lack of clarity around the variability and its source was one reason for Orzack’s skepticism with regards to the optimality of fig wasp sex ratio adjustment [8]. Comparisons of sex ratios within and among families under controlled conditions are required to establish whether variability originates from heritable differences or not. In order to describe the magnitude of over dispersion, studies should report hf (the ratio of residual deviance and the residual degrees of freedom minus (1) in addition to coefficients and significance.

## Problems and extensions

"future work will primarily be concerned with dotting i's and crossing t's." West and Herre [5] contemplating the future of sex ratio studies.

“the figures demand a much higher level of sex ratio than was observed. So … either the facts are misleading or the theory needs to be modified.”

Hamilton [16] confronting equation (1) with fig wasp data.

“further work will be required before a close match between theory and observation can be claimed.” 

Frank [18] confronting fig wasp data with his equations.

Despite the apparent good fit endorsed by the first quote [2, 4, 5], this view is not shared by all (2^nd^ and 3^rd^ quotes). In fact, many shortcomings have been pointed out and alternative explanations suggested [41, 50, 54, 58, 60, 100] and we summarize these here. First, we give some general information that have come to light on the mechanism resulting in sex ratio adjustment.

**Fig. 8:**
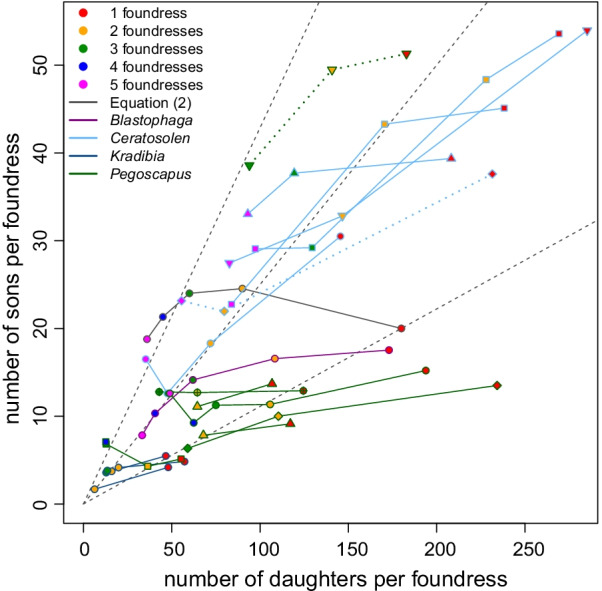
Species that reduce the number of daughters and sons remain constant or decrease. Seventeen data sets, each connected by a line, for 13 species with line and symbol outline colour indicating the genus and symbol fill indicating the foundress number: Symbol shapes are as follows: *Blastophaga nipponica* (circle), *Ceratosolen capensis* (circle), *C. fusciceps* (rombus), *C. gravelyi* (square), *C. marchali* (up triangle), *C. solmsi* (down triangle), *Kradibia tentacularis* (circle), *Pegoscapus aerumnosus* (circle), *P. herrei* (rombus), *P. jimenezi* (square), *P. longiceps* (circle with +), *P. silverstrii* (up triangle) and *Pegoscapus* sp. ex *Ficus crocata* (down triangle). Confidence intervals are not given as these are normally not reported. The grey line and circles indicate clutch compositions that would make Herre’s *n*_h_ assumption [17] and Eq. (2) correct at the same time for an imaginary single-foundress clutch size of 180 and *n*_h_ = 2. The dotted lines are for two species where number of sons and number of daughters were both divided by 2. Note that the scales of the axes differ. Details in Additional files 1 and 3

Experimental studies that interrupt foundresses' oviposition by killing foundresses have shown that foundresses use a slope strategy where most male eggs are laid first, followed by mostly female eggs [41, 64, 65, 68]. This implies that (1) smaller clutches will have a higher fraction of males as is commonly observed (13 species: [59, 62, 64, 66, 68, 69, 77, 79, 100] with *V. javana* being an exception [112]. (2) Females that lay smaller clutches, because they arrived later, or because they oviposit slower, or were less competitive, will automatically lay less female biased sex ratios which is optimal [29, 84]. (3) This strategy of laying daughters last (ladies-last) creates a situation where sex ratios will automatically become less female biased as foundress number increases when there is a limit on the total number of eggs that can be laid per fig [53, 83]. Indeed, this could explain the observation that the number of sons per foundress stays relatively constant when foundress number increases from one to two while the number of daughters decreases (this is the case for 7 of the 12 species illustrated in Fig. 8; *B. nipponica* [56], *C. gravelyi* [41, 77], *K. tentacularis* [64], *P. jimenezi* [19], *P. longiceps* [78], *P. silvestrii* [50, 78] and *Pegoscapus* sp. ex *Ficus crocata* [78]). In the four remaining species with two foundress data in Fig. 8, the number of daughters per mother is 55% or less in two foundress figs than one foundress figs. In these species the number of males are also reduced, but by less, suggesting that most male eggs, but not all, are laid first (*C. capensis* [53]*, C. fusciceps* [41], *P aerumnosus* [49], and *P herrei* [78]). The observation that sex ratios become male biased at unnaturally high foundress numbers [7, 41, 50] also suggests that this mechanism functions. Note that such male-biased ratios do not prove that sex ratio adjustment is not an adaptation to LMC as the situations are unusual or never arise.

**Fig. 9:**
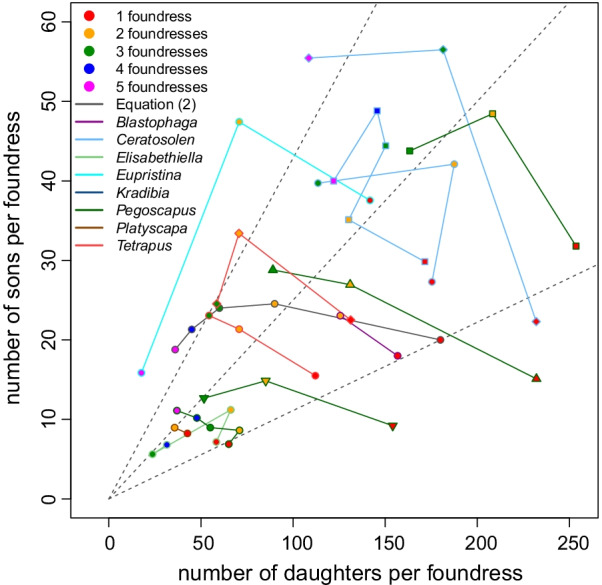
Species that increase the number of sons while the number of daughters remain constant or decrease. Thirteen data sets, each connected by a line, from 13 species with line and symbol outline colour indicating the genus and symbol fill indicating the foundress number. Symbol shapes are as follows: *B. nipponica* (circle), *C. fusciceps* (rombus), *C. galili* (circle), *C. solmsi* (square), *Elisabethiella baijnathi* (circle), *Eupristina koningsbergeri* (circle), *Pegoscapus franki* (circle), *P. lopesi* (square), *P. piceipes* (up triangle), *P. tonduzi* (down triangle), *Platyscapa awekei* (circle) *Tetrapus ecuadoranus* (circle) and *Tetrapus* sp. ex *Ficus insipida* (down triangle). Confidence intervals are not given as these are normally not reported. The grey line and circles indicate clutch compositions that would make Herre’s *n*_h_ assumption [17] and Eq. (2) correct at the same time for an imaginary single-foundress clutch size of 180 and *n*_h_ = 2. Note that the scales of the axes differ. Details in Additional files 1 and 3

The ladies-last effect will only result in sex ratio adjustments along the lines of LMC when females cannot lay all their eggs. Therefore, in figs where two or more foundresses can lay all their eggs, this effect is insufficient. Recent work [68, 70] suggests that some species combine the ladies-last effect with a facultative increase in the number of sons in figs with several foundresses as opposed to one foundress (this is the case for the 13 species illustrated in Fig. 9 (*B. nipponica* [57] *C. fusciceps* [79], *C. galili* [70], *C. solmsi* [68], *E. baijnathi* [53], *Eupristina koningsbergeri* [41], *Pegoscapus franki* [19], *P. lopesi* [78], *P. piceipes* [78], *P. tonduzi* [78], *Platyscapa awekei* [66], *Tetrapus ecuadoranus* [78] and *Tetrapus* sp. ex *Ficus insipida* [78]. We will refer to it as the dual mechanism. However, this dual mechanism is not always present in larger figs as *B. psenes* shows (Fig. S2; GLM with Poisson errors and an offset for foundress number, sons: *P* = 0.676, daughters: *P* = 0.631). This may be because *B. psenes* co-foundresses are often related [121].

### Too female biased and too little female bias

Even though sex ratios are female biased and increase with foundress numbers (Fig. 4), single foundress sex ratios seem to be not biased enough. Nagelkerke's [28] predictions are below all observed numbers. On the other hand, multifoundress figs are significantly more female biased than Eq. (2) predicts (taking averages for species and treating species as independent data, two foundresses: paired Wilcoxon signed rank test, V = 86, *P* = 0.035, 16 out of 24 species were lower; three foundresses: paired Wilcoxon signed rank test, V = 32, *P* = 0.002, 17 out of 20 species were lower). Wasps are more on target for two-foundress figs than for higher foundress numbers. The sex ratio of the mean two-foundress brood is 0.015 too low while the mean three-foundress brood is 0.043 too low. The fact that pollinating fig wasp tend to lay too many females (Fig. 4) is a common problem that has resulted in many alternative explanations. If we take into account that foundresses often leave figs after oviposition [85–87] the too-high female bias is potentially a bigger problem, though it may be compounded when individual figs actually contain two pollinating wasp species [21, 93, 122–129] (see point 3 below). We discuss explanations for the too highly female-biased ratios next:

1. Two suggestions of the importance of higher-level selection have been made. First, group selection at the level of the tree [16, 18, 19, 56]. In general wasp populations show very little genetic differentiation [130] and any group level selection will be very weak. This is therefore not a tenable general explanation. Second, group selection at the level of the fig may be important for some species. It is assumed that when females leave their natal fig, they join a large population of wind-dispersed conspecifics and will enter a small number of figs (by comparison). This is certainly the case for monoecious species where crops are synchronised within trees so that any one tree’s figs will be at the same developmental stage and where wasps ascend high above the canopy and then let themselves drift in the wind over large distances [131, 132]. As a result, chance will ensure that foundresses in the same fig are unrelated [17, 18]. In many dioecious species however, crops overlap on a single tree and wasps that leave from one fig are both likely to be related and to enter the same receptive fig [121, 133]. This limited dilution and mixing of unrelated wasps [121] should result in more female biased sex ratios [18, 30, 134]. While a general reduction of sex ratios is observed in two species [30, 56], it may be unrealistic to expect females to behave differently when they cofound with a sister as compared to when they cofound with an unrelated female. However, in species where females frequently cofound with sisters and unrelated females a facultative response can be expected. It is thus not surprising that neither *P. franki* [18] nor *C. solmsi* [26] responded, but surprising that it was not seen in *B. psenes* [30, 135]. Although *Diaziella yangi* (Sycoecinae, Chalcidoidea) is not from the pollinating lineage (Agaonidae, Chacidoidea), it enters figs to oviposit and LMC will apply. It is the only fig wasp species known to lay more female biased ratios when co-founders are related [136].
2. Unequal clutch sizes increases inbreeding and changes mating prospects. Increased inbreeding results in more female-biased sex ratios as does a strategy that does not take own clutch size into account [18]. When females take their own clutch size into consideration, no change is predicted to the brood sex ratio [30] (except for the inbreeding effect). Foundresses that enter simultaneously can show severe interference competition, from fighting lethally [42] to delaying and preventing oviposition by one foundress [43]. The mean clutch size of 11 *Pegoscapus* species [137] and several other wasps (Fig. 8) decreased as foundress number increased. Similarly, in 6 species, two wasps that entered simultaneously laid less eggs than two that entered sequentially [27, 37, 41, 60, 64, 112] while in 7 other species no such effect was seen [19, 53, 55, 66, 70]. The most extreme interference is when one/some fail to lay any eggs [75, 93]. Based on behavioural observations, the species studied by Hamilton [16], *P. aerumnosus*, engages in lethal fights, supposedly resulting in some foundresses laying no or few eggs [42] (Fig. 8). In addition nematodes may reduce infected females’ clutch sizes [138], although the effects of a necrophagous nematode may be insignificant [139]. Molecular techniques can help with identifying figs where one/some foundresses did not oviposit [93] or even determine exact clutch sizes [60, 66]. However, when only samples of a brood are genotyped, small contributions can be missed.
3. Initially it was believed that each fig harbours only one pollinating species [140]. However, it has become clear that individual figs of some species harbour more than one pollinating species [21, 93, 122–129]. When researchers were/are unaware that they are actually working on two species the counted foundress number will be inflated and the expected ratio higher than it should be. This can help to explain sex ratios that are too female-biased. Here molecular techniques can also help to disentangle the clutches of foundresses of the different species resulting in an improved fit [93]. Bizarrely, the wasps may on some occasions make the same mistake: *C. galili* foundresses inappropriately adjust their sex ratio in the presence of *C. arabicus* females [70].
4. Sex biased mortality will skew the secondary sex ratio away from the primary sex ratio. Li et al. [96] found that male larval mortality is substantially higher than female larval mortality in *C. fusciceps* (0.23 versus 0.04 (if the sex ratio was 0.16)). Given the consistently high levels of mortality, this mortality bias may be an important explanation of ratios being too female-biased. However, Galil and Eisikowitch [92] found that female *C. arabicus* are more prone to developmental mortality and the generality [71], within and among species, of Li et al.*’s* [96] finding needs to be established.
5. The optimal sex ratios during sequential oviposition are more female-biased than during simultaneous oviposition [18, 84]. Although the figs start to become unattractive and in some species the ostiole starts to become impenetrable once enough wasps oviposited [39, 40], this may allow additional females to enter after the first completed its oviposition [27, 41, 54, 57]. If sequential entry is common in naturally founded populations this could explain the too female-biased ratios and may explain single foundress sex ratios in addition (see below). Sequential entry can however not explain too female-biased sex ratios in experimental studies where females were entered in short succession [19], although simultaneous entry may be unnatural.
6. Males of haplodiploid taxa are only related to their daughters and have no male offspring. As a result one can expect these males to bias sex ratios in favour of females [16, 141]. There is however no evidence for an effect of autosomes on sex ratios by males in Hymenoptera [142]. If there is such an effect one can expect it to be mediated via factors in the males’ ejaculates, like in *Drosophila* [143]. If ejaculates contain substances that cause females to use more sperm, then we may expect sex ratios to increase with the number of matings. If the females are in control, no change in the ESS is expected. While the females of certain species only mate once (*K. tentacularis* [63]; *Alfonsiella* species *and Allotriozoon heterandromorphum* [144]; *Courtella gabonensis* and *Courtella camourensis* [145]), the females of some fig wasp species, particularly from the genus *Ceratosolen* mate multiple times [62, 146]. While one study found an effect of multiple matings on sex ratio, it was probably due to large females laying more eggs rather than them being mated more than once [62]. Multiple mating does not reduce the longevity of females [96]. If it reduced longevity, females may have laid smaller clutches, that would have been less biased due to the negative regression between sex ratio and clutch size [100].
7. Hamilton [16] and Frank [18] argued that if sex is determined by cytoplasmic elements the sex ratio would be very female biased because cytoplasmic elements are not transmitted by sperm. *Wolbachia* is such a cytoplasmically inherited parasite that skews sex ratios towards females in a variety of arthropods [147]. Even though *Wolbachia* are found in more than 80% of fig wasps, the average sex ratios of pollinating species with and without *Wolbachia* are not significantly different [148]. However, because sex ratios are largely skewed towards females and because a number of males are required to allow female emergence from the fig, there may be selection against cytoplasmic elements that would eliminate males.
8. When a ladies-last automatic adjustment approach is used, the sex ratios of higher foundress numbers will be too high [149]. Then the best strategy is a compromise between an overshoot at high foundress numbers and an undershoot at low foundress numbers [70]. However, the too female-bias increases, rather than decreases, at higher foundress numbers (Fig. 4).
9. In cases where there are normally only one foundress [7, 144, 150] it is unrealistic to expect even two foundress sex ratios to be accurately adjusted as this situation is simply experienced too seldomly. Rather, the strategy would be identical to that of single foundresses and bar a ladies-last effect, the clutch composition will be identical.
10. Moore et al. [60] argue that when a constant-male-first/ladies-last effect results in sex ratio adjustment, then foundress number is not the cue used to adjust sex ratios [58, 60, 149]. However, as the number of eggs carried by individual foundresses in their ovarioles relative to the number of available oviposition sites in a fig varies among wasps [43], figs [151, 152], crops and seasons, this mechanism of sex ratio response to foundress number cannot produce precise sex ratios. In addition, the foundress number cue may be time-dependent. Kinoshita [57] found that the first foundress only responds to a second if the second enters within half an hour from herself, but not after 4 h. When wasps do not perceive the cue, sex ratios will be too female biased. Point 5 overlaps with this suggestion.
11. In order to calculate *F* from *n*_h_ [17] two assumptions are made routinely. (1) Cofoundresses' clutches have similar sizes [7, 18] and (2) mating is random within a fig. Higher rates of sibmating than random [153] would result in sex ratios that appear too biased. However, mating may be more outbred than random rather than more inbred than random [94].

### Dispersal by adult wasps

Most pollinating wasps mate strictly locally as the standard LMC model assumes. Therefore, in most fig wasp species, Orzack's [9] mating structure blindspot does not apply. However, in some species male, and in some species female wasps disperse after or during oviposition and male dispersal changes the predictions. Male dispersal out of their natal fig and into another fig to mate has evolved at least twice [144]. In *Alfonsiella pipithiensis* it reduces the proportion of sibmating by 6.5%, which should reduce the female bias [48]. The species’s female bias is indeed lower than non-dispersing species, but more than predicted [48].

It is assumed that males disperse and mate randomly within a fig. Together with an assumption of equal clutch sizes it allows the probability of sibmating to be worked out from harmonic mean foundress numbers [17]. In addition, it allows the calculation of fitness using equation (S1), which is a core assumption for the relationship between foundress number and sex ratio. Male dispersal between figs invalidates these assumptions because excess males disperse to other figs [150, 154].

On completion of oviposition the foundresses of many species disperse out of the fig they oviposited in [85–88, 155]. Some of these females may even enter a second fig to oviposit there too; others simply die on the outside of the fig. Despite re-emergence, females still lay their eggs in groups within which LMC applies. However, re-emergence invalidates the observation approach to wasp number determination as that approach will systematically underestimate the number of females that contributed to the fig. Re-emergence should result in sex ratios that appear not to be female biased enough (the opposite is common).

### Single foundresses

Nagelkerke's [28] LMC model's predictions for single foundress sex ratios are more female biased than observed. These models were developed for small clutches where it is realistic to assume that a single male can mate all females in a patch. Rather than seeing this as evidence for a lack of fit to Nagelkerke's [28] predictions, single foundress sex ratios have by default been considered the ratios that give just enough males. In fig wasps just enough males mean enough sons should be lain to fertilize all the daughters and release them from their figs [16]. We call this limit the baseline. If a fig contains too few males several things can happen, they may fail to chew an exit tunnel resulting in so-called coffin figs where wasps exit their galls but cannot escape the fig before they die. Alternatively, the hole may be chewed, but not all females may be mated (or have received sufficient sperm). In haplodiploids this results in constrained allocation where such females will lay sons only or mostly sons [156]. In fig wasps constrained allocation is typically excluded from sex ratio data sets but it does affect the sex ratio of a fraction of figs (1.6% [75]; 2.6% [116]; 3.7% [68]; 4.8% [48]; 5.3% [19]; 8% [70]; very rare [92]). It is also known that a small fraction of females leaving figs are not mated [72–74]. It is naïve to think that the baseline will be a specific number beyond which coffin figs will never occur [48], rather as a fig contains more males, they will be better able to excavate an exit tunnel [76]. In smaller figs a single male may be sufficient to chew the tunnel, but in larger figs several males may be necessary and they work together synergistically [76]. As many as 9 *A. pipithiensis* males can fail to chew an exit tunnel out of *Ficus craterostoma* figs [48] while a single *Nigeriella excavata* and *P. awekei* male can chew a tunnel into their host’s small figs [144]. In *K. tentacularis* a single male will succeed 63% of the time and this number rises to 89% for 4 males [76]. However, this measure fails to detect females whose sex allocation will be constrained because of no-mating or too few sperm [19, 48–50, 68, 70, 72–75, 116].

Another approach to estimate the baseline is to look at the limit of sex ratios in large clutches. Larger clutches have less variance in sex ratio and the threat of maleless figs due to chance becomes less (Fig. 7; [100]). We can therefore expect such large clutches to approach the baseline requirement of males most accurately. On average this is 1 male to 24 females [100], which is well below the mean of 0.11. This number will certainly vary from species to species with males’ mating ability, fig sizes but also with wasp biology.

**Fig. 10:**
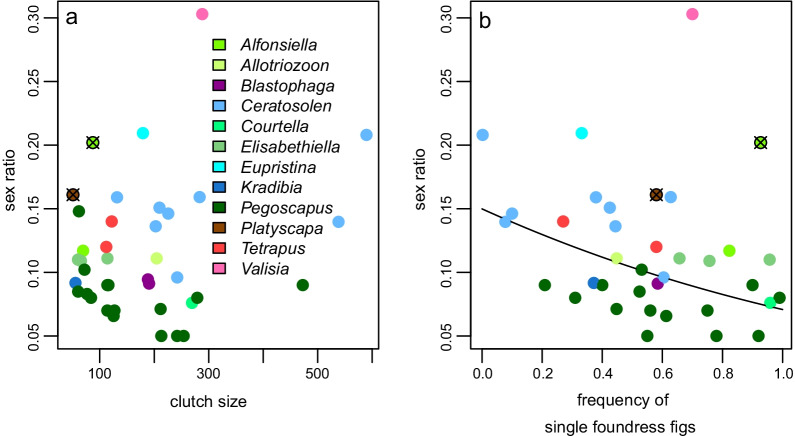
Single foundress sex ratio variation. **a** Observed mean sex ratios against mean clutch sizes of 39 species. **b** Observed mean sex ratios against the frequency of single foundress figs in 36 species. Each genus is colour coded and the colour legend applies to a and b. The two points encircled in black and with an x indicate the two species with dispersing males, *A. pipithiensis* and *P*. *awekei*. The line in b indicates the back-transformed regression on the log-odds of the sex ratios and frequency of single foundress frequencies and excludes the two dispersing species (*P* < 0.019). Details in Additional files 1 and 4

Several alternative explanations have been given for single foundress sex ratios and we look at these next. The first explanation is to avoid too few males. Indeed, West et al. [73] found that species with smaller clutches have more virgin females. Because the variance in sex ratios increases as clutch size decreases, smaller clutches should have higher sex ratios (Fig. 10a). This idea is not in line with data for among species comparisons because larger mean clutches are not more female biased (linear regression on log-odds for mean within species single-foundress fig sex-ratio against mean clutch size, with two dispersing species and *V. javana* excluded: *P* = 0.261). The lack of importance of clutch size may be because their mean clutch sizes are typically larger than 20 and a slope strategy will mean that maleless figs are very unlikely. This suggests that the trend that single foundress sex ratios increase as clutch sizes decrease within species [100] is probably the result of a slope strategy that is terminated at different time points (Fig. 5b). As theory would suggest, the two dispersing species have higher sex ratios, but they also have small clutch sizes (Fig. 10).

Second, given that a second foundress can enter after the first foundress died or completed oviposition [41, 54, 55, 57, 59, 68, 77, 112], the first foundress needs to produce enough males to hedge her bets against a second and even more foundresses entering later [54]. The model expectation for two sequential foundresses have been worked out by Suzuki and Iwasa [84]. They found the non-cooperative equilibrium of strategies for a situation where a proportion *p* of patches have two foundresses and the remaining (1—*p*) have only one foundress. They showed that if *N*_*1*_ = first foundress’s clutch size, *N*_2_ = second foundress’s clutch size, setting *B* = *N*_2_/*N*_1_, and assuming egg survival is independent of the total number of eggs that were laid and replacing some of their notation with our equivalents, they found first female’s strategy, $r_{1}^{*}$, the second female’s strategy, $r_{2}^{*}$, and the resulting brood sex ratio, $r_{b}^{*}$, as:

3a $$r_{1}^{*} = \frac{1 + F}{{1 + 2F}} \cdot \frac{1 + B}{2} \cdot \frac{{p^{2} }}{{\left( {1 + p} \right)^{2} }}$$

3b $$r_{2}^{*} = \frac{1 + F}{{1 + 2F}} \cdot \frac{1 + B}{{2B}} \cdot \frac{p}{{\left( {1 + p} \right)^{2} }}$$

3c $$r_{b}^{*} = \frac{1 + F}{{2\left( {1 + 2F} \right)}} \cdot \frac{p}{1 + p}$$

**Fig. 11:**
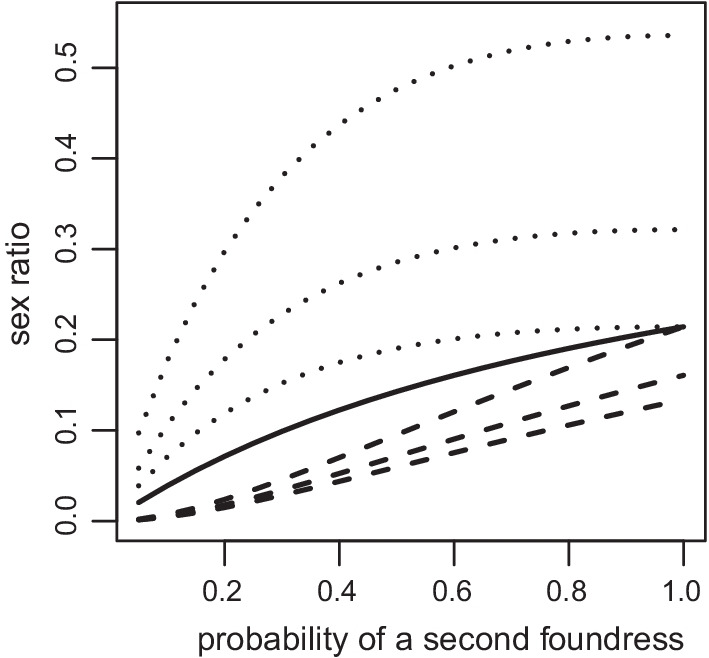
The non-cooperative equilibrium sex ratios for two sequential foundresses. The first foundress's (dashed) and second foundress’s sex ratio (dotted) for when *B* = *1*, *B* = 0.5 and *B* = 0.25 (bottom to top for dotted lines and top to bottom for dashed lines; *B* is the relative clutch size of the second female compared to the first). The brood sex ratio for any relative clutch sizes (solid) is also given. These were calculated for when *F* = 0.2

Equations (3a) and (3c) reach a maximum equal to Eq. (2)’s prediction for two foundresses when *p* = 1. However, if there are any one-foundress figs as is common in fig wasps, i.e. *p* < 1, then the brood sex ratio will be lower in the sequential scenario than in a simultaneous situation (Fig. 11). We would thus expect that single foundress’s sex ratios should decrease as the frequency of single foundress figs increases as has been observed [7, 54]. This trend is confirmed in this updated data set (Fig. 10b; linear regression, explaining the log-odds as a function of single foundress frequency, *P* < 0.001). A phylogenetic regression would be more appropriate because *Ceratosolen* wasps tend to have less frequent single foundress figs while *Pegoscapus* tends to have more. Despite Herre et al.'s [81] claim of a lack of phylogenetic constraint a controlled comparison suggested that there is phylogenetic inertia in fig wasp sex ratios [157]. Indeed, lone *Ceratosolen* foundresses lay proportionally many more males than lone *Pegoscapus* foundresses (Figs. 8, 9). After mating, male *Ceratosolen* collectively cut an exit hole from the fig, a feature that may relate to the coriaceous fig wall of subgenus *Sycomorus* figs, and emerge en masse providing food for the ants that are often present on subgenus *Sycomorus* figs, protecting the emerging females [158, 159]. On the contrary, only few *Pegoscapus* males participate in cutting the exit hole [160, 161].

Interestingly, sequential clutches’s combined sex ratio is unaffected by the relative clutch sizes (Eq. (3c)). The combined clutches', or brood sex ratio only depends on the frequency at which a second foundress is expected to arrive (*p*) and increases with *p*. Individuals with a smaller clutch must produce a substantially higher sex ratio (Fig. 11). This would be achieved automatically with a slope oviposition strategy. In the jewel wasp *Nasonia vitripennis* (not a fig wasp), this is achieved by second females laying at least seven sons before laying any daughters whereas first females lay at least three daughters before any sons are laid [109].

Third, ladies-last models optimize the number of males that should be laid first by exact females, given the foundress number distribution, oviposition site limitations and mortality rates [149]. These models assume simultaneous oviposition and that all male eggs are laid first. Because these models have no facultative sex ratio adjustment, foundresses have to evolve a number of male strategy that gives the best mean fitness over all foundress number situations and this number of males can be determined in single foundress figs. Indeed, the resultant single foundress sex ratios of six species were well described by this approach [149]. Similar results should be obtained if a slope strategy is used in ladies-last models and we suspect it will give predictions like Fig. 8.

#### More-than-one foundress figs

We already discussed the observation that the most common problem is that sex ratios are too female-biased and we offered eleven reasons why this may be so. We ruled out only four of these as unlikely, all the other explanations may contribute. A focus on sex ratios rather than on the number of sons and daughters has clouded our vision of fig wasp sex ratio data (compare Fig. 4 to Figs. 8 and 9). Even though the numbers of sons and daughters are normally not tested statistically we can discern two clear patterns across 25 species (Figs. 8, 9). In the first group (Fig. 8), consisting of 15 data sets from 12 species, the number of daughters produced per mother in two foundress figs decreased compared to single foundress figs. The number of sons either remained the same or dropped by less than the number of daughters. In the second group (Fig. 9) consisting of 13 data sets from 13 species, two-foundress mothers produced more sons per mother than single foundress mothers. The number of daughters they produce either decreased or remained the same. A lack of raw data prevents us from testing these differences in numbers of sons and daughters statistically (but see [70]). One may expect each species to fall in one pattern. However, three species had data sets that fell in both groups. It is possible that these splits are the result of a lack of statistical tests. But, it can also be that due to differences between trees, figs or even wasps differing significantly in size or that the cues used by wasps were inadvertently destroyed or created. Such environmental variation can explain why species may fall in both patterns. Only three data sets did not fall into these two patterns (Fig. S2) and these are all cases where their sex ratios were not significantly different between one and two foundresses (Fig. 4). Apart from *B. psenes*, a lack of raw counts does not allow statistical testing. It is noteworthy that even though *B. psenes* mothers laid more daughters and sons in two foundress figs, neither of these were statistically significant (GLM with Poisson errors and an offset for foundress number, sons: *P* = 0.676, daughters: *P* = 0.631). This suggests no response, which may be in line with the host fig's large figs and foundresses often being related.

More details on these two patterns illustrate that a slope strategy is used and not an exact strategy. Two-foundress figs of 10 species had 25% more sons per mother than single foundress figs. The number of sons per mother was 25% less in two-foundress figs of 7 species in group one. In all these cases the number of daughters decreased by a greater percentage. The frequent reduction in number of sons in two-foundress figs compared to single-foundress figs suggests that a slope strategy rather than an exact strategy [149] is used. These data suggest that pollinating fig wasps have a very simple strategies that result in near optimal sex ratios. 1) Lay mostly male eggs first, followed by mostly female eggs. Natural selection should optimize the intercept and slope of a slope strategy, taking into account the expected number of future foundresses, the expected number of co-foundresses, the expected number of oviposition sites, and the expected mortality. (2) In some species where a ladies-last effect will be insufficient and multi-foundress figs frequent, lay more male eggs when a second or more foundresses are present. The magnitude of the change will depend on the expected number of future foundresses/co-foundresses and the expected number of oviposition sites. In *C. solmsi* females increase the number of sons in three- compared to two foundress figs as well [68]. (3) In species with re-emergence, the absence of other females holds less information modulating point 2. However, the scarcity of flowers with two eggs [37], means that females can get information regarding previous females from cues other than the actual female, such as oviposition markers.

How much of these strategies are the result of selection for optimal sex ratios is not clear. The traits that are potentially under selection include, (1) having a slope strategy that entail laying mostly males first, (2) the intercept and slope of such a slope strategy, (3) changes in the slope and intersection when there are different foundress numbers. These traits should be the focus of future investigations. Sometimes foundresses will incorporate additional information, such as the clutch size of co-foundresses [66], but this may be the exception rather than the rule. The current data do not allow one to draw any conclusions about sequential as opposed to simultaneous entry. Since observational studies may be sequential rather than simultaneous, only experimental studies can investigate this difference.

## Conclusion: are pollinating fig wasps’ sex ratios adaptations?

" It is ... time to comfortably acknowledge our failures and to rescue important facts from being forgotten. " Orzack [9]

Williams [162] argued that the designation “adaptation” should be reserved for traits that have become fixed in populations due to natural selection for a specific outcome. The brood compositions of 24 species (Figs. 8, 9), the sex ratio trends of 25 species (Fig. 4) and the lack of conformity to assumptions that link foundress number to *F* illustrate that the initial extension of LMC theory does not fit data and it did not answer Hamilton’s plea [16]. It is clear that sex ratios do in general respond to LMC in being female biased. This conclusion is bolstered by the clear fitness advantage of biased ratios. On the other hand, the evidence that selection shaped conditional sex ratio adjustments at the time of oviposition, depending on local foundress number in a fig as reflected by Eq. (2), is not very convincing. This is in part due to the small fitness benefits, but also due to more convincing alternative explanations, serious breaches of model assumptions and consistent failures to explain observed data. Therefore, the amalgam of evidence suggests that Eq. (2) and its associated assumptions seems to ascribe more precision to fig wasps than exist with not a single species fitting both the predictions (Fig. 4) and assumptions (Figs. 8, 9) of Eq. (2). In fact, consistent and significant biases in observed ratios suggest that the model consistently neglect important parameter(s). In testing models it is important to restate that a failure to reject a predicted value, (e.g. [17]) does not constitute proof of a fit. In studies on singe species, comparisons of alternative explanations can help to identify missing parameters [66, 163]. In addition to this shortfall, the lack of data on the oviposition behaviour of individual females means that the level of testing and the causal level of models are mismatched. The lack of individual data means that alternative models cannot be compared (e.g. [17, 18, 20, 29, 84]).

Showing that a trait increases fitness is just a first step to showing that it is an adaptation because the increase may be coincidental. By studying convergent evolution of fig-entering wasps belonging to non-pollinating lineages and of pollinating wasps belonging to the Agaonidae [164, 165] and studying trait evolution in this speciose taxon using phylogenies [166] one could answer the role selection played. For instance, from a phylogenetic perspective, *Kradibia*, *Ceratosolen* and *Eupristina* had their last common ancestor more than 70 million year ago [167]. Therefore, the observation that the slope strategy is present in all three genera suggests that it has been maintained for 70 million years, suggesting that it may be an adaptation that has been fine-tuned by natural selection. Comparative phylogenetic studies on aspects of the slope are required to determine which traits could be considered adaptations.

A ladies (or mostly ladies)-last strategy amounts to wasps using their own clutch size as a cue [60]. Such a slope strategy is surprisingly versatile, increasing fitness in a variety of situations: sequential entry, smaller and larger relative clutches. Such a slope strategy can result in passive adjustments in line with theory. However, the passive nature of adjustments does not mean that natural selection did not favour a slope strategy, nor that the slope strategy has not been shaped by natural selection. It only means that Eq. (2) and its derivation do not capture the dynamics of natural selection. Natural selection may however still have played an important role. The observation that the sequence of laying specific sex eggs seem to be reversed from many other Hymenoptera [105] suggests that the existence of a slope that begins with males may be an adaptation.

Some studies have aimed at rejecting Eq. (2) by showing that wasps fail to respond optimally in unusual situations. Such approaches are mistaken. For example, testing sex ratios of related foundresses in species where foundresses are always unrelated [18] cannot show that selection failed, nor that is was successful because selection is absent. Similarly, testing foundress numbers far in excess of normal numbers [41] cannot clarify the role that natural selection may or may not have played. These experimental manipulations may however clarify mechanisms as in the case of re-introductions of *C. fusciceps* discussed above.

Any explanation of sex ratios will rely on fitness calculated by equation (S1) and will therefore be affected by haplodiploid’s asymmetric inclusive fitness and by LMC through mating opportunities. As a result, the direction and even the magnitude of predictions will be similar for most models. Deciding which model is appropriate must be decided on the basis of whether assumptions are met and by comparing models’ abilities to predict observations [163].

By only focusing narrowly on Eq. (2) important basic biology has been overlooked. The most important of these is probably what causes variation in offspring number? Is it caused by (1) fighting, (2) different egg loads, (3) different wasp ages, (4) different arrival times, (5) different nematode loads or (6) different fig sizes. Wasp offspring number can vary substantially between trees and even between figs. Experiments or observations on trees in botanical gardens that may be watered or fertilized, may yield very different results from natural trees. This may be particularly important when stress may affect wasp size [168] and hence wasp fecundity [43]. These differences can also be the result of figs that can remain receptive for several days, and brood size and offspring size may be affected by the timing of foundress entries [39, 69, 169]. The overlap in oviposition should also be strongly affected by basic fig biology, with oviposition tending to be more simultaneous in species with smaller figs than large figs. In smaller figs, ostiole closure is likely to be shortly after first entry, preventing females from entering long after each other. In addition to considering the basic biology of figs, this review suggests that research should urgently address whether the high larval mortality is sex-biased or not, how often oviposition is sequential rather than simultaneous, and how brood size and composition vary with foundress numbers. Figures like Figs. 8 and 9 are useful to clarify mean wasp behaviour and a combination with molecular techniques can allow a focus on individual behaviour. Because sex ratio is likely to be an emergent trait [9] with selection shaping variables of the slope, rather than the sex ratio itself, research must quantify the slope rather than sex ratios.

While fig wasps provide wonderful models to investigate sex ratio strategies under diversified conditions, they also present serious limitations. One is that homogenous genetic lines are difficult or even impossible to breed and it is therefore unlikely that genetic variation in sex ratios can be distinguished from environmental effects. Environmental variation in clutch and fig size will not affect the optimality of a binomial strategy with a probability of drawing a male egg adjusted to local conditions, but it will bedevil a slope strategy. A slope strategy will invariably be mismatched with the current clutch size and fig size and historical variation in *F*. Such environmental variation will continually change the predicted fitness rewards and costs. It is thus a pipe dream to assume that homogeneous selection will over time result in a single fine-tuned strategy.

Nevertheless, fig wasps are wonderful [7].

## Supplementary Information

**Additional file 1.** Supplementary text and figures. Explanation of methods, derivation of ESS sex ratio, analysis of wasp size and supplementary figures.

**Additional file 2.** Figure 4 data. Observed (± 95% CI) and expected sex ratios in 36 studies of 25 species.

**Additional file 3.** Figures 8 and 9 data. Clutch composition in 33 studies on 25 species.

**Additional file 4.** Figure 10 data. Single Foundress sex ratios, fraction of single foundress figs and clutch size of 39 species.

## Data Availability

The data presented in this manuscript were extracted from published literature or obtained from the authors when the raw data was not available in the original publication. All of the presented data are provided in supplementary information files.

## Abbreviations

*B*: *= N*_2_/*N*_1_, The clutch size of a second foundress relative to that of the first
bin: Binomial strategy
Brood: The wasps offspring eclosing from one fig irrespective of the number of mothers
Clutch: The wasp offspring of a single mother
E2 females: Females that produce Eq. (2) sex ratios
ESS: Evolutionary stable strategy
ex: Exact strategy
*F*: Inbreeding coefficient
fig wasp: Meaning pollinating fig wasp
gw: Gall wasp
hf: Heterogeneity factor
IA females: Females that produce an invariant, but adjusted sex ratio of 3/14
LMC: Local mate competition
*n*: Foundress number
*n*_h_: Harmonic mean foundress number
*N*_1_: Clutch size of first foundress
*N*_2_: Clutch size of second foundress
*p*: Proportion of patches with two foundresses
pkw: Parasitoid and kleptoparasitoid wasps
pw: Pollinating wasp
*r*^*^: ESS sex ratio for a fixed foundress number
*r*_1_*: Non-cooperative sex ratio of the first foundress
*r*_2_*: Non-cooperative sex ratio of the second foundress
*r*_b_*: Brood sex ratio of two sequential non-cooperative foundresses
*r*_n_*: ESS sex ratio for a female in an *n* foundress patch
*s*: Probability that a female is sibmated
sl: Slope strategy
UA females: Females that produce an unadjusted 50:50 sex ratio

## References

[1] Hamilton WD (1996). Narrow roads of gene land.

[2] West SA, Herre EA, Sheldon BC (2000). The benefits of allocating sex. Science.

[3] Charnov EL (1982). The theory of sex allocation.

[4] West SA (2009). Sex allocation.

[5] West SA, Herre EA, Hardy ICW (2002). Using sex ratios: why bother?. Sex ratios: concepts and research methods.

[6] Seger J, Stubblefield JW, Hardy ICW (2002). Sex ratio theory. Sex ratios: concepts and research methods.

[7] Herre EA, West SA, Cook JM, Compton SG, Kjellberg F, Choe J, Crespi BJ (1997). Fig-associated wasps: pollinators and parasites, sex-ratio adjustment and male polymorphism, population structure and its consequences. The evolution of mating systems in insects and arachnids.

[8] Orzack SH (1995). Test of optimality models: reply from S. H. Orzack. Trends Ecol Evol.

[9] Orzack SH, Hardy ICW (2002). Using sex ratios: the past and the future. Sex ratios: concepts and research methods.

[10] Orzack SH, Sober E (1994). Optimality models and the test of adaptationism. Am Nat.

[11] Orzack SH (1990). The comparative biology of second sex ratio evolution within a natural population of a parasitic wasp *Nasonia vitripennis*. Genetics.

[12] Orzack SH, Parker ED (1990). Genetic variation for sex ratio traits within a natural population. Genetics.

[13] Hamilton WD (1967). Extraordinary sex ratios. Science.

[14] Taylor PD (1981). Intra-sex and inter-sex sibling interactions as sex ratio determinants. Nature.

[15] Green RF, Gordh G, Hawkins BA (1982). Precise sex ratios in highly inbred parasitic wasps. Am Nat.

[16] Hamilton WD, Blum MS, Blum NA (1979). Wingless and fighting males in fig wasps and other insects. Reproductive competition, mate choice and sexual selection in insects.

[17] Herre EA (1985). Sex ratio adjustment in fig wasps. Science.

[18] Frank SA (1985). Hierarchical selection theory and sex ratios. II on applying the theory, and a test with fig wasps. Evolution..

[19] Frank SA (1983). Theoretical and empirical studies of sex ratios, mainly in fig wasps.

[20] Nunney L, Luck RF (1988). Factors influencing the optimum sex ratio in a structured population. Theor Popul Biol.

[21] Michaloud G, Michaloud-Pelletier S, Wiebes JT, Berg CC (1985). The co-occurrence of two pollinating species of fig wasp and one species of fig. K Ned Akad Van Wet Ser C.

[22] Compton SG (1993). One way to be a fig. Afr Entomol.

[23] Patel A, Hossaert-McKey M (2000). Components of reproductive success in two dioecious fig species *Ficus exasperata* and *Ficus hispida*. Ecology.

[24] Peng Y-Q, Yang D-R, Zhou F, Zhang. Pollination biology of *Ficus auriculata* Lour. in tropical rainforest of Xishuangbanna. Acta Phytoecol Sin. 2003;77:111–7.

[25] Peng YQ, Compton SG, Yang DR (2010). The reproductive success of *Ficus altissima* and its pollinator in a strongly seasonal environment: Xishuangbanna. Southwestern China Plant Ecol.

[26] Hu HY, Ma GC, Niu LM, Fu YG, Peng ZQ, Huang DW (2010). The effects of relatedness on offspring sex ratio in pollinating fig wasps. Evol Ecol Res.

[27] Deng X-X, Wu L-F, Yu H (2017). Influence factors on offspring reproduction of pollinator in a highly species-specific mutualism of *Ficus*. J Trop Subtrop Bot.

[28] Nagelkerke CJ (1996). Discrete clutch sizes, local mate competition and the evolution of precise sex allocation. Theor Popul Biol.

[29] Yamaguchi Y (1985). Sex ratio of an aphid subject to local mate competition with variable maternal condition. Nature.

[30] Kjellberg F. La stratégie reproductive du figuier (*Ficus carica* L.) et de son pollinisateur (*Blastophaga psenes* L.) un exemple de coévolution. [Thèse de Docteur Ingénieur]. [Paris]: INAPG; 1983.

[31] Düsing C (1884). Die regulierung des geschlechtsverhältnisses bei der vermehrung der menschen, tiere, und pflanzen. Jen Zeitschrift für Naturwissenschaft.

[32] Düsing C (1884). Die Regulierung des Geschlechtsverhältnisses.

[33] Cooper L, Bunnefeld L, Hearn J, Cook JM, Lohse K, Stone GN (2020). Low-coverage genomic data resolve the population divergence and gene flow history of an Australian rain forest fig wasp. Mol Ecol.

[34] Kjellberg F, Jousselin E, Hossaert-McKey M, Rasplus J-Y. Biology, ecology and evolution of Ficus (Moraceae) pollinating wasps (Chalcidoidea, Agaonidae). In: Raman A, Schaefer CV, Withers TM, editors. Biology, ecology and evolution of gall-inducing arthropods. New Hampshire; 2005. p. 539–71.

[35] Pereira RAS, De Pádua TS, Kjellberg F (2007). An inquiline fig wasp using seeds as a resource for small male production: A potential first step for the evolution of new feeding habits?. Biol J Linn Soc.

[36] Jousselin E, Hossaert-Mckey M, Vernet D, Kjellberg F (2001). Egg deposition patterns of fig pollinating wasps: Implications for studies on the stability of the mutualism. Ecol Entomol.

[37] Ghana S, Suleman N, Compton SG (2012). Factors influencing realized sex ratios in fig wasps: double oviposition and larval mortalities. J Insect Behav.

[38] Kjellberg F, Doumesche B, Bronstein J (1988). Longevity of a fig wasp (*Blastophaga psenes*). Proc K Ned Akad Van Wetenscappen Ser C- Biol Med Sci.

[39] Khadari B, Gibernau M, Anstett MC, Kjellberg F, Hossaert-McKey M (1995). When figs wait for pollinators: the length of fig receptivity. Am J Bot.

[40] Hu H-Y, Jiang Z-F, Niu L-M, Fu Y-G, Peng Z-Q, Huang D-W (2009). Different stimuli reduce attraction to pollinators in male and female figs in the dioecious fig *Ficus hispida*. Biotropica.

[41] Wang R-W, Sun B-F, He J-Z, Dunn DW (2015). Non-quantitative adjustment of offspring sex ratios in pollinating fig wasps. Sci Rep.

[42] Dunn DW, Jandér KC, Lamas AG, Pereira RAS (2015). Mortal combat and competition for oviposition sites in female pollinating fig wasps. Behav Ecol.

[43] Moore JC, Greeff JM. Resource defence in female pollinating fig wasps: Two’s a contest, three’s a crowd. Anim Behav. 2003;66.

[44] Wang RW, Ridley J, Sun BF, Zheng Q, Dunn DW, Cook J (2009). Interference competition and high temperatures reduce the virulence of fig wasps and stabilize a fig-wasp mutualism. PLoS ONE.

[45] Jandér KC, Herre EA (2016). Host sanctions in Panamanian *Ficus* are likely based on selective resource allocation. Am J Bot.

[46] Jandér KC, Herre EA, Simms EL (2012). Precision of host sanctions in the fig tree-fig wasp mutualism: consequences for uncooperative symbionts. Ecol Lett.

[47] Herre EA (1987). Optimality, plasticity and selective regime in fig wasp sex ratios. Nature.

[48] Greeff JM (2002). Mating system and sex ratios of a pollinating fig wasp with dispersing males. Proc R Soc Lond B.

[49] Pereira RAS, Prado AP (2006). Effect of local mate competition on fig wasp sex ratios. Braz J Biol.

[50] Ramírez-Benavides W, Monge-Nájera J, Chavarría JB (2009). Sex ratio in two species of *Pegoscapus* wasps (Hymenoptera : Agaonidae) that develop in figs: can wasps do mathematics, or play sex ratio games ?. Int J Trop Biol.

[51] Cruaud A, Jabbour-Zahab R, Genson G, Cruaud C, Couloux A, Kjellberg F (2010). Laying the foundations for a new classification of Agaonidae (Hymenoptera: Chalcidoidea), a multilocus phylogenetic approach. Cladistics.

[52] Elias LG, Lino-Neto J, Pereira RAS (2018). Oogenesis and ovarian morphology in pollinating and non-pollinating fig wasps: evidence from adult and immature stages. Invertebr Reprod Dev.

[53] Nefdt RJC (1989). Interactions between fig wasps and their host figs.

[54] Greeff JM, Compton SG (1996). Sequential oviposition and optimal sex ratios in pollinating fig wasps. Ecol Entomol.

[55] Bronstein JL, Vernet D, Hossaert-McKey M (1998). Do fig wasps interfere with each other during oviposition?. Entomol Exp Appl.

[56] Kinoshita M, Kasuya E, Yahara T (1998). More highly female-biased sex ratio in the fig wasp, *Blastophaga nipponica* Grandi (Agaonidae). Res Popul Ecol.

[57] Kinoshita M, Kasuya E, Yahara T (2002). Effects of time-dependent competition for oviposition sites on clutch sizes and offspring sex ratios in a fig wasp. Oikos.

[58] Kathuria P, Greeff JM, Compton SG, Ganeshaiah KN (1999). What fig wasp sex ratios may or may not tell us about sex allocation strategies. Oikos.

[59] Moore JC, Compton SG, Hatcher MJ, Dunn AM (2002). Quantitative tests of sex ratio models in a pollinating fig wasp. Anim Behav.

[60] Moore JC, Zavodna M, Compton SG, Gilmartin PM (2005). Sex ratio strategies and the evolution of cue use. Proc R Soc B.

[61] Peng Y-Q, Yang D-R, Wang Q-Y (2005). Adjustment and stabilization of sex ratio in *Ceratosolen solmsi marchali*. Acta Ecol Sin.

[62] Peng Y, Zhang Y, Compton SG, Yang D (2014). Fig wasps from the centre of figs have more chances to mate, more offspring and more female-biased offspring sex ratios. Anim Behav.

[63] Zavodna M, Compton SG, Raja S, Gilmartin PM, van Damme JMM (2005). Do fig wasps produce mixed paternity clutches ?. J Insect Behav.

[64] Raja S, Suleman N, Compton SG, Moore JC (2008). The mechanism of sex ratio adjustment in a pollinating fig wasp. Proc R Soc B.

[65] Sun B-F, Wang R-W, Hu Z (2009). Ovipositing pattern of the fig wasps and its effect on the offspring sex ratio. Zool Res.

[66] Greeff JM, Newman DVK (2010). Testing models of facultative sex ratio adjustment in the pollinating fig wasp *Platyscapa awekei*. Evolution.

[67] Yan X, Peng Y-Q, Yang D-R (2012). Spatial distribution patterns of three fig wasps on *Ficus semicordata*: How non-pollinators affect pollinator’s sex ratio. Acta Ecol Sin.

[68] Hu HY, Chen ZZ, Jiang ZF, Huang DW, Niu LM, Fu YG (2013). Pollinating fig wasp *Ceratosolen solmsi* adjusts the offspring sex ratio to other foundresses. Insect Sci.

[69] Liu C, Yang D, Compton SG, Peng Y (2013). Larger fig wasps are more careful about which figs to enter – with good reason. PLoS ONE..

[70] Greeff JM, Pentz K, Warren M. The efficacy of natural selection in producing optimal sex ratio adjustments in a fig wasp species. Proc R Soc. 2020;287:20201377. 10.1098/rspb.2020.1377 PMC7542795 32900311

[71] Jansen-González S, de Teixeira SP, Pereira RAS (2012). Mutualism from the inside: coordinated development of plant and insect in an active pollinating fig wasp. Arthropod Plant Interact..

[72] Godfray HCJ (1988). Virginity in haplodiploid populations : a study on fig wasps. Ecol Entomol.

[73] West SA, Herre EA, Compton SG, Godfray HCJ, Cook JM (1997). A comparative study of virginity in fig wasps. Anim Behav.

[74] West SA, Compton SG, Vincent SL, Herre EA, Cook JM (1997). Virginity in haplodiploid populations: a comparison of estimation methods. Ecol Entomol.

[75] Zavodna M, Knapp SM, Compton SG, Arens P, Vosman B, Van Dijk PJ (2007). Reconstruction of fig wasp mating structure: How many mothers share a fig?. Ecol Entomol.

[76] Suleman N, Raja S, Compton SG (2012). Only pollinator fig wasps have males that collaborate to release their females from figs of an Asian fig tree. Biol Lett.

[77] Zhang Y, Peng Y-Q, Yang D (2014). Effects of foundress number, foundress entry interval and non-pollinating wasps on clutch size and offspring sex ratio of pollinating fig wasps (Hymenoptera: Agaonidae). Acta Entomol Sin.

[78] Herre EA. PhD: Sex ratio adjustment in thirteen species of Panamanian fig wasps. [Iowa]: The University of Iowa; 1988.

[79] Chendi MA, Yang L-Y, Liu T (2019). The primary research of sex ratio mechanism of pollinating fig wasp in *Ficus racemosa*. J Yunnan Agric Univ.

[80] West SA, Herre EA (1998). Stabilizing selection and variance in fig wasp sex ratios. Evolution.

[81] Herre EA, Machado CA, West SA, Orzack SH, Sober E (2001). Selective regime and fig wasp sex ratios. Adaptionism and optimality.

[82] Anstett MC, Kjellberg F, Cefe JLB (1996). Waiting for wasps: consequences for the pollination dynamics of *Ficus pertusa* L. J Biogeogr.

[83] Werren JH (1980). Sex ratio evolution under local mate competition in a parasitic wasp. Science.

[84] Suzuki Y, Iwasa Y (1980). A sex ratio theory of gregarious parasitoids. Res Popul Ecol.

[85] Gibernau M, Hossaert-McKey M, Anstett MC, Kjellberg F (1996). Consequences of protecting flowers in a fig: a one-way trip for pollinators?. J Biogeogr.

[86] Moore JC, Dunn AM, Compton SG, Hatcher MJ (2003). Foundress re-emergence and fig permeability in fig tree-wasp mutualisms. J Evol Biol.

[87] Hu H-Y, Niu L-M, Ma G-C, Fu Y-G, Peng Z-Q, Huang D-W (2010). Permeability of receptive fig fruits and its effects on the re-emergence behaviour of pollinators. Ecol Entomol.

[88] Mohd Hatta SK, Quinnell RJ, Idris AG, Compton SG. Making the most of your pollinators: an epiphytic tree encourages its pollinators to roam between figs. Ecol Evol. 2021;11:6371–80. 10.1002/ece3.7488 PMC8207429 34141224

[89] Yu H, Compton SG (2012). Moving your sons to safety: galls containing male fig wasps expand into the centre of figs, away from enemies. Plos ONE..

[90] Zhang X-W, Chen C, Wang R-W, Kjellberg F (2020). The cost of parasitism: High larval developmental mortality following attacks by a parasitoid fig wasp on a fig pollinating wasp. Acta Oecol..

[91] Al-Beidh S, Dunn DW, Power SA, Cook JM (2012). Parasites and mutualism function: measuring enemy-free space in a fig–pollinator symbiosis. Oikos.

[92] Galil J, Eisikowitch D (1971). Studies on mutualistic symbiosis between syconia and sycophilous wasps in monoecious figs. New Phytol.

[93] Molbo D, Machado CA, Sevenster JG, Keller L, Herre EA (2003). Cryptic species of fig-pollinating wasps: implications for the evolution of the fig-wasp mutualism, sex allocation, and precision of adaptation. Proc Natl Acad Sci.

[94] Zavodna M, Compton SG, Biere A, Gilmartin PM, Van Damme JMM (2005). Putting your sons in the right place: the spatial distribution of fig wasp offspring inside figs. Ecol Entomol.

[95] Tian E-W, Yu H, Zhang D-Y, Nason JD (2011). Development of microsatellite loci for *Blastophaga javana* (Agaonidae), the pollinating wasp of *Ficus hirta* (Moraceae). Am J Bot.

[96] Li ZT, Peng YQ, Wen XL, Jandér KC (2016). Selective resource allocation may promote a sex ratio in pollinator fig wasps more beneficial for the host tree. Sci Rep.

[97] Zhang X-W, Dunn DW, Wang RW (2020). Egg load is a cue for offspring sex ratio adjustment in a fig-pollinating wasp with male-eggs-first sex allocation. J Evol Biol.

[98] Gould SJ, Vrba ES (1982). Exaptation—a missing term in the science of form. Paleobiology.

[99] Wilson K, Hardy ICW, Hardy ICW (2002). Statistical analysis of sex ratios: an introduction. Sex ratios: concepts and research methods.

[100] Kjellberg F, Bronstein JL, Ginkel GV, Greeff JM (2005). Clutch size : a major sex ratio determinant in fig pollinating wasps ?. C R Biol.

[101] Greeff JM. How serious is the assumption of no phenotypic variation in optimality models? A sex ratio example. S Afr J Sci. 1998;94:269–70.

[102] Otto SP, Day T (2007). A biologist’s guide to mathematical modelling in ecology and evolution.

[103] Waage JK, Ming NS. The reproductive strategy of a parasitic wasp: I. optimal progeny and sex allocation in Trichogramma evanescens. J Anim Ecol. 1984;53:401–15.

[104] Waage JK. Family planning in parasitoids: adaptive patterns of progeny and sex allocation. In: Waage JK, Greathead D, editors. Insect Parasitoids: 13th Symposium of the Royal Entomological Society of London: Academic Press; 1986. p. 63–95.

[105] King BH (1987). Offspring sex ratios in parasitoid wasps. Q Rev Biol.

[106] Flanders SE (1946). The mechanisms of sex-ratio regulation in the (parasitic) Hymenoptera. Insectes Soc.

[107] Flanders SE (1946). Control of sex and sex-limited polymorphism in the Hymenoptera. Q Rev Biol.

[108] Wylie HG (1973). Control of egg fertilization by *Nasonia vitripennis* (Hymenoptera: Pteromalidae) when laying on parasitized house fly pupae. Can Entomol.

[109] King BH (1993). Sequence of offspring sex production in the parasitoid wasp, *Nasonia vitripennis*, in response to unparasitized versus parasitized hosts. Anim Behav.

[110] Tarachai Y, Compton SG, Trisonthi C (2008). The benefits of pollination for fig wasps. Symbiosis.

[111] Peng Y-Q, Yang D-R, Duan Z-B, Deng X-B (2005). Reproductive components of *Ficus hispida* and its pollinator. Acta Phytoecol Sin.

[112] Yu H, Compton SG, Wu L (2018). Spatial variation in pollinator gall failure within figs of the gynodioecious *Ficus hirta*. Acta Oecol.

[113] Zhao JB, Peng YQ, Quinnell RJ, Compton SG, Yang DR (2014). A switch from mutualist to exploiter is reflected in smaller egg loads and increased larval mortalities in a “cheater” fig wasp. Acta Oecol.

[114] Nefdt RJC, Compton SG (1996). Regulation of seed and pollinator production in the fig-fig wasp mutualism. J Anim Ecol.

[115] Jousselin E, Kjellberg F, Herre EA (2004). Flower specialization in a passively pollinated monoecious fig: a question of style and stigma?. Int J Plant Sci.

[116] Chen Y, Chen X, Wu W, Wang Z, Lu B (2013). Community structure and species biodiversity of fig wasps in syconia of Ficus superba Miq. Var. japonica Miq. in Fuzhou. Acta Ecol Sin..

[117] Suleman N, Raja S, Compton SG (2013). Parasitism of a pollinator fig wasp: mortalities are higher in figs with more pollinators, but are not related to local densities of figs. Ecol Entomol.

[118] Raja S, Suleman N, Quinnell RJ, Compton SG. Interactions between pollinator and non-pollinator fig wasps: Correlations between their numbers can be misleading. Entomol Sci. 2015;18.

[119] Pereira RAS, Prado AP (2005). Non-pollinating wasps distort the sex ratio of pollinating fig wasps. Oikos.

[120] West SA, Herre EA (1994). The ecology of the New World fig-parasitizing wasps Idarnes and implications for the evolution of the fig-pollinator mutualism. Proc R Soc Lond B.

[121] Kjellberg F, Gouyon P-H, Ibrahim M, Raymond M, Valdeyron G (1987). The stability of the symbiosis between dioecious figs and their pollinators: a study of Ficus carica L. and Blastophaga psenes L. Evolution..

[122] Ramírez BW (1970). Host specificity of fig wasps (Agaonidae). Evolution.

[123] Rasplus J-Y. The one-to-one species specificity of the *Ficus*-Agaoninae mutualism: how casual? In: van der Maesen LJG, van der Burgt XM, van Medenbach de Rooy JM, editors. The biodiversity of African plants. Dordrecht: Springer Netherlands; 1996. p. 639–49.

[124] Compton SG, Grehan K, Van Noort S (2009). A fig crop pollinated by three or more species of Agaonid fig wasps. Afr Entomol.

[125] Moe AM, Rossi DR, Weiblen GD. Pollinator sharing in dioecious figs (*Ficus*: Moraceae). Biol J Linn Soc. 2011;546–58.

[126] Cornille A, Underhill JG, Cruaud A, Johnson SD, Tolley KA, Kjellberg F (2012). Floral volatiles, pollinator sharing and diversification in the fig – wasp mutualism : insights from F*icus natalensis,* and its two wasp pollinators (South Africa). Proc R Soc B.

[127] Darwell CT, Cook JM (2014). Molecular species delimitation of a symbiotic fig-pollinating wasp species complex reveals extreme deviation from reciprocal partner specificity. BMC Evol Biol.

[128] Rodriguez LJ, Bain A, Chou L, Conchou L, Cruaud A, Gonzales R, et al. Diversification and spatial structuring in the mutualism between *Ficus septica* and its pollinating wasps in insular South East Asia. BMC Evolutionary Biology; 2017;1–12. 10.1186/s12862-017-1034-8 PMC5576367 28851272

[129] Yu H, Tian E, Zheng L, Deng X, Cheng Y, Chen L (2019). Multiple parapatric pollinators have radiated across a continental fig tree displaying clinal genetic variation. Mol Ecol.

[130] Tian E, Nason JD, MacHado CA, Zheng L, Yu H, Kjellberg F. Lack of genetic isolation by distance, similar genetic structuring but different demographic histories in a fig-pollinating wasp mutualism. Mol Ecol. 2015;24. 10.1111/mec.13438 26518361

[131] Compton SG, Ellwood MDF, Davis AJ, Welch K (2000). The flight heights of Chalcid wasps (Hymenoptera, Chalcidoidea) in a lowland Bornean rain forest: fig wasps are the high fliers. Acta Soc Zool Bohemicae.

[132] Harrison RD, Rasplus J-Y (2006). Dispersal of fig pollinators in Asian tropical rain forests. J Trop Ecol.

[133] Kjellberg F, Lesne A. *Ficus carica* and its pollination [Internet]. 2020. Available from: https://hal.archives-ouvertes.fr/hal-02516888

[134] Gardner A, Hardy ICW (2020). Adjustment of sex allocation to co-foundress number and kinship under local mate competition: an inclusive-fitness analysis. J Evol Biol.

[135] Megia R. Ettude du sex-ratio du Blastophage: effect de l’apparentement et controle par le figuier [Diplome]. University Paris-Sud Orsay; 1989.

[136] Song B, Peng Y-Q, Yang D-R (2008). The role of foundress relatedness in the offspring sex ratio of fig wasp *Diaziella yangi* (Hymenoptera : Pteromalidae). Acta Entomol Sin.

[137] Herre EA (1989). Coevolution of reproductive characteristics in 12 species of New World figs and their pollinator wasps. Experientia.

[138] Herre EA (1993). Population structure and the evolution of virulence in nematode parasites of fig wasps. Science.

[139] Van Goor J, Piatscheck F, Houston DD, Nason JD (2018). Figs, pollinators, and parasites: a longitudinal study of the effects of nematode infection on fig wasp fitness. Acta Oecol.

[140] Wiebes JT (1979). Co-evolution of figs and their insect pollinators. Annu Rev Ecol Syst.

[141] Hawkes P (1992). Sex-ratio stability and male-female conflict over sex-ratio control in hymenopteran parasitoids. S Afr J Sci.

[142] Shuker DM, Sykes EM, Browning LE, Beukeboom LW, West SA (2006). Male influence on sex allocation in the parasitoid wasp *Nasonia vitripennis*. Behav Ecol Sociobiol.

[143] Rice WR (1996). Sexually antagonistic male adaptation triggered by experimental arrest of female evolution. Nature.

[144] Greeff JM, van Noort S, Rasplus J-Y, Kjellberg F (2003). Dispersal and fighting in male pollinating fig wasps. C R Biol.

[145] Michaloud G, Devez AR. Pollination ecology in tropical figs - a case of Mutualism. Service du Film de Recherche Scientifique, (SFRS) 92170 Vanves, France; 1982.

[146] Murray MG (1990). Comparative morphology and mate competition of flightless male fig wasps. Anim Behav.

[147] Werren JH, Baldo L, Clark ME (2008). *Wolbachia*: master manipulators of invertebrate biology. Nat Rev Microbiol.

[148] Ahmed MZ, Greyvenstein OFC, Erasmus JC, Welch JJ, Greeff JM (2013). Consistently high incidence of *Wolbachia* in global fig wasp communities. Ecol Entomol.

[149] Chung N, Pienaar J, Greeff JM (2019). Evolutionary stable sex ratios with non-facultative male-eggs first sex allocation in fig wasps. Oikos.

[150] Nelson RM, Greeff JM (2009). Evolution of the scale and manner of brother competition in pollinating fig wasps. Anim Behav.

[151] Bronstein JL, Hossaert-McKey M (1996). Variation in reproductive success within a subtropical fig/pollinator mutualism. J Biogeogr.

[152] Anstett MC, Bronstein JL, Hossaert-McKey M (1996). Resource allocation: a conflict in the fig/fig wasp mutualism?. J Evol Biol.

[153] Frank SA (1985). Are mating and mate competition by the fig wasp *Pegoscapus asssuetus* (Agaonidae) random within a fig?. Biotropica.

[154] Moore JC, Loggenberg A, Greeff JM (2006). Kin competition promotes dispersal in a male pollinating fig wasp. Biol Lett.

[155] Suleman N, Raja S, Quinnell RJ, Compton SG (2013). Putting your eggs in several baskets : oviposition in a wasp that walks between several figs. Entomol Exp Appl.

[156] Godfray HCJ (1990). The causes and consequences of constrained sex allocation in haplodiploid animals. J Evol Biol.

[157] Hansen TF, Orzack SH (2005). Assessing current adaptation and phylogenetic inertia as explanations of trait evolution: the need for controlled comparisons. Evolution.

[158] Harrison RD (2014). Ecology of a fig ant-plant. Acta Oecol.

[159] Zachariades C, Schatz B, Compton SG (2010). Wasp emergence from the figs of *Ficus sur*: characteristics and predation by ants. Trop Zool.

[160] Ramírez BW (1970). Taxonomic and biological studies of neotropical fig wasps (Hymeopter: Agaonidae). Univ Kans Sci Bull.

[161] Frank SA. The behavior and morphology of the fig wasps *Pegoscapus assuetus* and *P. jimenezi*: desriptions and suggested behavioral characters for phylogenetic studies. Psyche (Camb. Mass.). 1984;91:289–308.

[162] Williams GC (1966). Adaptation and natural selection: a critique of some current evolutionary thought.

[163] Hilborn R, Mangel M (1997). The ecological detectives: confronting models with data.

[164] Jousselin E, Rasplus J-Y, Kjellberg F (2001). Shift to mutualism in parasitic lineages of the fig/fig wasp interaction. Oikos.

[165] Kjellberg F, Proffit M (2016). Tracking the elusive history of diversification in plant–herbivorous insect–parasitoid food webs: insights from figs and fig wasps. Mol Ecol.

[166] Hansen TF, Pienaar J, Orzack SH (2008). A comparative method for studying adaptation to a randomly evolving environment. Evolution.

[167] Cruaud A, Rønsted N, Chantarasuwan B, Chou LS, Clement WL, Couloux A (2012). An extreme case of plant-insect codiversification: Figs and fig-pollinating wasps. Syst Biol.

[168] Jandér KC, Dafoe A, Herre EA (2016). Fitness reduction for uncooperative fig wasps through reduced offspring size: A third component of host sanctions. Ecology.

[169] Zhang Y, Yang DR, Peng YQ, Compton SG (2012). Costs of inflorescence longevity for an Asian fig tree and its pollinator. Evol Ecol.

