# Supplementary material for: Pollinating fig wasps’ simple solutions to complex sex ratio problems: a review
Source: Front Zool. 2022 Jan 12;19:3. doi: 10.1186/s12983-021-00447-4 (PMC8756665; doi:10.1186/s12983-021-00447-4)
Supplement: Supplementary file 1 — Additional file 1. Supplementary text and figures. Explanation of methods, derivation of ESS sex ratio, analysis of wasp size and supplementary figures. [file 12983_2021_447_MOESM1_ESM.docx]

Pollinating fig wasps’ simple solutions to complex sex ratio problems: a review

Jaco M. Greeff^1,*^ & Finn Kjellberg^2^

^1^Department of Biochemistry, Genetics and Microbiology, University of Pretoria, Pretoria, South Africa

^2^CEFE, CNRS, Univ Montpellier, EPHE, IRD,

* Correspondence:

Jaco M. Greeff

jaco.greeff@up.ac.za

# Supplementary Text

## Methods

The source and method of data extraction for data presented in Figs. 4, 8, 9 and 10 are given in supplementary files. The most accurate estimates of sex ratios, number of sons and daughters were made given the available material. When data were presented in tables or in the text, these were used. If not given, numbers of sons were calculated by multiplying the mean sex ratios with mean clutch sizes. When data were only presented in figures, the authors were emailed to ask for the specific estimates. Failing this, figures were scanned and digitized using Plot Digitizer [1]. Data were considered when it was based on 3 or more figs, when broods were more than 30 eggs large, and when sex ratios were unconstrained. Data were recorded for up to 5 foundresses.

We preferred estimates of the inbreeding coefficient that were based on molecular data. This was typically presented as an *F*_IS_ or were calculated as *F* = (*H*_e_ - *H*_o_)/*H*_e_ when only *H*_e_ = expected heterozygosity and *H*_o_ = the observed heterozygosity were given. When *n*_h_ was given, *F* = 1/(4*n*_h_ - 3). The frequency of single foundresses was taken directly from tables, measured from figures or obtained from authors.

All calculations, statistics and graphs were done in R version 4.1.0 [2]. To test if observed sex ratios were significantly lower than predicted sex ratios, a Wilcoxon paired rank test was used. Raw data was only available for 12 of the 36 data sets. Therefore, generalized linear models (GLMs) could not be fitted to all data. The method by which 95% CIs were calculated is given in Fig. 4's legend. Numbers of sons and daughters were tested using GLMs with Poisson errors and and offset for foundress number.

## Derivation of the standard inclusive fitness model

Consider a fig with *n* foundresses that all lay equal clutch sizes of *c* eggs. Focus on the fitness of a mutant female that produce a sex ratio of *r*_m_ when all other mothers lay a sex ratio of *r*. Her fitness is the sum of her fitness through daughters and sons. The fitness through each sex is not simply the number of each sex because their genetic value must be incorporated in a standard inclusive fitness approach and son’s fitness will depend on their mating opportunities [3–5]. In more detail: this number of offspring has to be multiplied by the reproductive value of the sex offspring (*v*_m_ for males and *v*_f_ for females) and the relatedness of that sex offspring to the mother (*R*_m_ and *R*_f_ respectively) [6]. *v*_m_ = *v*_f_/2 [7] and while *R*_m_= 1, in inbred haplodiploids *R*_f_ depends on *F* and the relatedness of daughters to mothers is *R*_f_ = (1 + 3*F*)/(2(1 + *F*)) = 1/(2 - *s*), with *s* = probability that a female is sibmated and *F* = s/(4 - 3*s*). The ratio of the genetic value of males to females is thus (1 + *F*):(1 + 3*F*) or (2 - *s*):2. Finally, while for daughters, we only need to count the number of daughters (*c*(1 - *r*_m_)), we need to calculate the expected number of matings of her *cr*_m_ sons. This is the total number of daughters in a fig that is given by ((*n* - 1)*c*(1 - *r*) + *c*(1 - *r*_m_)) divided by the total number of males in a fig, which is given by ((*n* - 1)*cr* + *cr*_m_). If we put all of this together we get the focal female’s fitness *w*_m_ (*m* subscript for mutant, not male) as:

*w*_m_ = *c*(1 - *r*_m_)*v*_f_*R*_f_ + *cr*_m_*v*_m_*R*_m_((*n* - 1)(1 - *r*) + (1 - *r*_m_))/((*n* - 1)*r* + *r*_m_) equation (S1)

This equation can be used to calculate the fitness of any sex ratio. If the clutch sizes are not identical then (*n* - 1)(1 - *r*) should be replaced by the number of daughters produced by other females and (*n* - 1)*r* by the number of sons produced by the other females. Therefore the assumption of clutch size being the same for all females is not crucial. If we are prepared to assume equal clutch sizes and that equation (S1) can be differentiated with respect to sex ratio, then we can obtain the ESS sex ratio for *n* foundresses as, *r*_n_*, by setting the derivative equal to zero, setting *r*_m_ = *r* = *r*_n_* and solving for *r*_n_*, giving:

*r*_n_* = $\frac{n-1}{n}\frac{v_{\text{m}}R_{\text{m}}}{v_{\text{m}}R_{\text{m}}+v_{\text{f}}R_{\text{f}}}=\frac{n-1}{n}\cdot\frac{1}{2}\cdot\frac{1+F}{1+2F}$ equation (S2)

## Competition does not affect wasp size

It is standard to believe that developing wasps do not compete amongst each other for resources. In fact, there is the impression that at least in some species more developing wasps are more effective at triggering the flow of nutrients than figs with fewer wasps. There is however no direct evidence for this point of view. One example of indirect evidence showing that wasp number has no effect on total available resources comes from reconsidering tree FC6 (*Ficus carica*, hosting *Blastophaga psenes*) for which Tayou [8] recorded female wasp size (wing length for 10 individuals) and the total brood size for 29 figs that contained 6 related (15) or 6 unrelated (14) foundresses. If there was competition one would expect the mean wasp size to decrease and the standard deviation of wasp size to increase as brood size increased. A linear model explaining mean wasp size as a function of foundress relatedness and brood size found no effect of either explanatory variable (df = 25 after deleting one influential outlier, relatedness: coefficient = -0.163, *P* = 0.605 brood size: coefficient < -0.001, *P* = 0.217). Similarly, a linear model explaining the standard deviation of wasp size as a function of foundress relatedness and brood size found no effect of either explanatory variable (df = 26, relatedness: coefficient = 0.124, *P* = 0.651 brood size: coefficient < -0.001, *P* = 0.189). There is thus no evidence for an effect, but data is required to confirm this widely held notion.

# Supplementary Figures

**Figure S1**. Same as Fig. 6, but clutch size = 28, therefore c = 0.90 and a = -0.35708525.

**
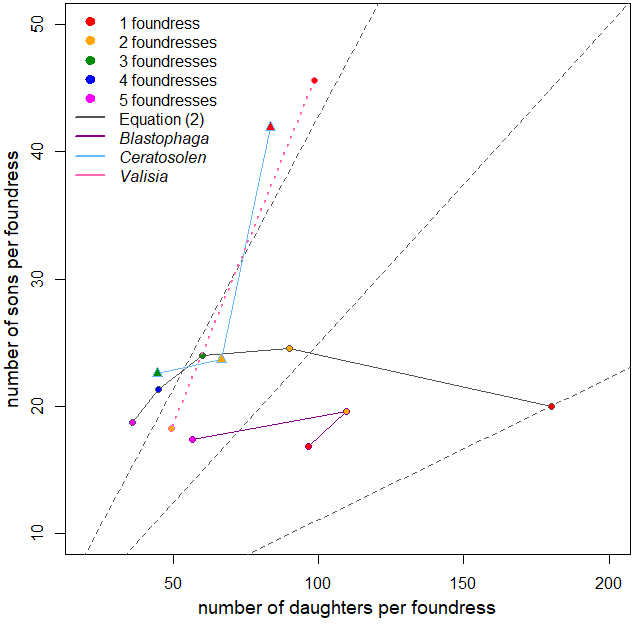
**

**Figure S2**. Species/experiments that did not fit the two typical patterns. Three data sets, each connected by a line with its colour and the symbol outline colour indicating the genus and symbol fill indicating the foundress number. Symbol shapes are as follows: *B. psenes* (circle), *Ceratosolen marchali* (upward triangle) and *Valisia javana* (circle). Confidence intervals are not given as these are normally not reported. The grey line and circles indicate clutch compositions that would make Herre’s *n*_h_ assumption [9] and equation (2) correct at the same time for an imaginary single-foundress clutch size of 200 and *n*_h_ = 2. The dotted lines are for one species where number of sons and number of daughters were both divided by 2. Details in Additional files 3. Note that the scales of the axes differ.

# References

1. Huwaldt JA. PlotDigitizer [Internet]. 2015. Available from: http://plotdigitizer.sourceforge.net

2. R Core Team. R: A language and environment for statistical computing. Viena: R Foundation for Statistical Computing; 2018.

3. Hamilton WD. Wingless and fighting males in fig wasps and other insects. In: Blum MS, Blum NA, editors. Reproductive Competition, Mate Choice and Sexual Selection in Insects. New York: Academic Press; 1979. p. 167–220.

4. Suzuki Y, Iwasa Y. A sex ratio theory of gregarious parasitoids. Res Popul Ecol. 1980;22:366–82.

5. Seger J, Stubblefield JW. Sex ratio theory. In: Hardy ICW, editor. Sex Ratios: Concepts and Research Methods. Cambridge: Cambridge University Press; 2002. p. 2–25.

6. Crozier RH, Pamilo P. Evolution of social insect colonies: sex allocation and kin selection. Oxford, New York: Oxford University Press; 1996.

7. Price GR. Selection and covariance. Nature, 1970;227:520–1.

8. Tayou A. Etude du Mutualisme *Ficus*-Pollinisateur: sex ratio et transport du pollen. Institut National Agronomique Paris-Grignon; 1991.

9. Herre EA. Sex ratio adjustment in fig wasps. Science. 1985;228:896–8.
